# Toward efficient multiple-site incorporation of unnatural amino acids using cell-free translation system

**DOI:** 10.1016/j.synbio.2021.12.007

**Published:** 2021-12-23

**Authors:** Jiaqi Hou, Xinjie Chen, Nan Jiang, Yanan Wang, Yi Cui, Lianju Ma, Ying Lin, Yuan Lu

**Affiliations:** a College of Life Sciences, Shenyang Normal University, Shenyang, 110034, China; b Key Laboratory of Industrial Biocatalysis, Ministry of Education, Department of Chemical Engineering, Tsinghua University, Beijing, 100084, China; c Guangdong Key Laboratory of Fermentation and Enzyme Engineering, School of Biology and Biological Engineering, South China University of Technology, Guangzhou, 510006, China

**Keywords:** Cell-free protein synthesis, Orthogonal translation system, Unnatural protein, Unnatural amino acid, Terminal codon suppression

## Abstract

Amber suppression has been widely used to incorporate unnatural amino acids (UNAAs) with unique structures or functional side-chain groups into specific sites of the target protein, which expands the scope of protein-coding chemistry. However, this traditional strategy does not allow multiple-site incorporation of different UNAAs into a single protein, which limits the development of unnatural proteins. To address this challenge, the suppression method using multiple termination codons (TAG, TAA or TGA) was proposed, and cell-free unnatural protein synthesis (CFUPS) system was employed. By the analysis of incorporating 3 different UNAAs (p-propargyloxy-l-phenylalanine, p-azyl-phenylalanine and L-4-Iodophenylalanine) and mass spectrometry, the simultaneous usage of the codons TAG and TAA were suggested for better multiple-site UNAA incorporation. The CFUPS conditions were further optimized for better UNAA incorporation efficiency, including the orthogonal translation system (OTS) components, magnesium ions, and the redox environment. This study established a CFUPS approach based on multiple termination codon suppression to achieve efficient and precise incorporation of different types of UNAAs, thereby synthesizing unnatural proteins with novel physicochemical functions.

## 1 Introduction

In nature, proteins constructed with 20 kinds of standard amino acids can have various structures and functions to fully maintain life, but these natural proteins are no longer sufficient to meet the research needs of protein engineering and functional protein production [1]. The incorporation of unnatural amino acid (UNAA) with novel side-chain groups can endow unnatural proteins with new chemical properties, structures, and functions [[1], [2], [3]]. At present, more than 150 UNAAs have been site-specifically incorporated into proteins [4,5]. UNAA incorporation methods mainly include global suppression based on natural translation system, termination codon suppression based on orthogonal translation system (OTS), code shift suppression, meaningful codon redistribution, and unnatural base pair [6]. Among three termination codons(TAG, TAA, TGA), because the termination codon TAG is the least used in *E. coli*, it is most widely used in the UNAA incorporation [7]. The termination codon suppression method is powerful and effective, and it has successfully incorporated various types of UNAAs specifically into the proteins [8]. For example, Natasha et al. used a genomically-recoded strain of *Escherichia coli* with a flexible TAG codon to produce site-specific phosphoserine-containing proteins, with purities approaching 90% [9]. Lajoie et al. knocked out the *prfA* gene that terminated translation and mutated all TAG termination codons to synonymous TAA termination codons in *E. coli* MG1655 to redistribute the TAG translation function, which effectively increased the UNAA incorporation efficiency [10]. In all UNAA incorporation methods, the termination codon suppression method has been the most widely used.

Currently, the synthesis of unnatural proteins is mainly dependent on the cellular system. However, the intracellular metabolic pathways are complex, and the target protein synthesis is not the main intracellular reaction, which results in low yield and cumbersome purification steps. Besides, UNAAs are not easy to pass through the cell membrane barrier, resulting in a low UNAA incorporation efficiency and toxic substances that affect the normal growth of cells. At the same time, because of the limitations of cell membranes, the controllability of cell responses is weak. All of these have led to lower efficiency of UNAA incorporation *in vivo*, especially in the multiple-site UNAA incorporation [2,[10], [11], [12]]. As a result, cell-free protein synthesis (CFPS) emerges as a new unnatural protein synthesis technology. Compared with intracellular expression, the cell-free system is simple to operate, resistant to cytotoxicity, flexible, and efficient, and it can overcome the limitations of cell membranes [13]. At present, the cell-free unnatural protein synthesis (CFUPS) system has become an effective unnatural protein synthesis platform. The ideal method is to use the modified OTS components, including UNAA and its orthogonal tRNA/orthogonal aminoacyl-tRNA synthetase (aaRS) pairs, which can covalently load UNAA onto the suppressor tRNA via acylation of aaRS [1]. In this way, add OTS components to a cell-free system for synthesizing unnatural proteins, which can provide unnatural protein novel physicochemical properties to achieve more functions, such as improving pharmacokinetics, cancer treatment, vaccine development, proteomics, and protein engineering [2,14,15].

In recent years, breakthroughs have been made in the UNAAs incorporation by termination codon suppression method in the CFUPS system. Hong et al. achieved the incorporation of p-propargyloxy-l-phenylalanine (pPaF) by knocking out release factor 1 (RF1), which could otherwise terminate translation in a cell-free system of *E. coli*, and the yield increased 2.5-fold [16]. However, up-regulation of the natural suppression mechanism promotes the formation of truncated products, especially for multiple-site incorporation experiments [10]. Jewett's group used strain engineering to effectively solve this problem. The CFUPS platform was developed by exploiting multiplex genome engineering to enhance extract performance by functionally inactivating negative effectors for accurate incorporation efficiency of p-acetyl-l-phenylalanine (≥98%) [17]. However, the incorporation of a single type of UNAA only using one termination codon, in one or more sites, in a protein of interest limits the possibilities for genetic code expansion. Being able to incorporate multiple different UNAAs may allow biosynthesizing cyclic proteins, many different simultaneous site-specific post-translational modifications, and protein labeling with different fluorophores [18]. Therefore, the use of TAA and TGA termination codon suppression methods to incorporate UNAAs has been studied. Ozerps group realized the combination of TAG termination codon suppression and TAA termination codon suppression in a cell-free system to combine two different types of UNAA p-azyl-phenylalanine (pAzF) and propargyl-l-lysine (PrK) incorporated into sfGFP [18]. Although some progress has been made in the incorporation of various UNAAs in CFUPS, the types and sites of UNAA incorporation are still limited and need to be further expanded. Furthermore, UNAA's incorporation efficiency in target proteins was mostly achieved by removing the competition of termination factors and improving the aminoacylation efficiency of the orthogonal tRNA. Based on these research advances, optimizing the cell-free system could be considered as a way to overcome enzyme inefficiencies by controlling the components and environment at precise ratios to further improve the UNAA's incorporation efficiency [17].

**Fig. 1:**
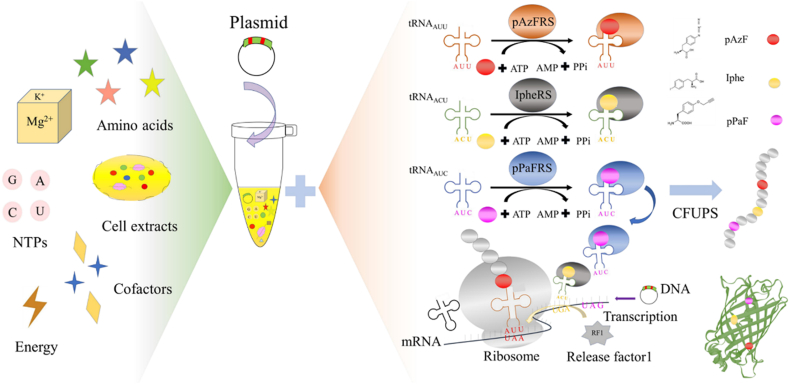
Overview of workflow for the UNAA incorporation in sfGFP based on the CFUPS system.

Therefore, this study proposed a precise strategy for the site-specific incorporation of two UNAAs using two kinds of termination codons in a single recombinant protein based on the CFUPS system (Fig. 1). Three groups of OTSs components were designed, in which pPaF, tRNA_CUA_ and pPaFRS were used for TAG suppression, pAzF, tRNA_UUA_ and pAzFRS were used for TAA suppression, and L-4-Iodophenylalanine (Iphe), tRNA_UCA_ and IpheRS were used for TGA suppression. By mass spectrometry, TAG and TAA termination codon suppression methods with the highest incorporation efficiency were employed for multiple-site UNAA incorporation study. To further improve the incorporation efficiency of UNAAs, the OTS components, magnesium ions (Mg^2+^) and redox environment of CFUPS were optimized. Finally, the yield of sfGFP reached 2.9 mg/mL, which was 1.7 times more than before [17]. Together with findings, suggested that two UNAAs were effectively incorporated in a single SFGFP based on the CFUPS system, which provided novel structures and functions for unnatural proteins.

## 2 Materials and methods

### 2.1 Strains, UNAAs, plasmids and primers

A total of three strains were used in this study, including *E. coli* DH10B, C321.ΔA [9] (Addgene #68306) and *E. coli* BL21(DE3). DH10B is suitable for various gene recombination experiments. The C321.ΔA is a genomically recoded organism in which all of the genomic TAG stop codons were mutated into TAA. This strain not only tolerated the deletion of RF1 but also eliminated all RF1-knockout-associated growth defects observed in partially recoded strains. The properties of C321.ΔA suggested that this strain would provide the ideal setting for UNAA incorporation, offering a completely open and assignable UAG codon in a background that did not suffer from the low purity and compromised fitness observed in other RF1-knockout strains [18]. *E. coli* BL21(DE3) is mainly applicable to the high levels of the protein expression of T7 promoter expression vector.

p-propargyloxy-l-phenylalanine (pPaF), p-azyl-phenylalanine (pAzF) and L-4-Iodophenylalanine (Iphe) were purchased from Sigma. The structural formulas of the three UNAAs were shown in Fig. 1. For the incorporation of multiple UNAAs into one protein, three different OTSs were required. pPaF OTS and pAzF OTS were selected according to the research of Swartz group previously [19]. L-phe OTS were used to incorporate UNAA into multiple UUU phenylalanine (Phe) codons in a site-specific manner [20]. DNA plasmids used in CFUPS were obtained from the culture of *E. coli* DH10B strain (Biomed Biotechnology, China) using Plasmid Mini kits (Omega Bio-Tek, America). All plasmids used in this experiment were sequence-verified. All linear PCR products (tRNA) were amplified with Pfu high-fidelity DNA polymerase (Beyotime Biotechnology, China). All primers, tRNA sequences and synthetic aaRSs were purchased from GENEWIZ Biotechnology with no modifications. All primer sequences could be found in Table S1.

### 2.2 Expression template design

The aaRS sequences were located between RBS and T7 termination on the pET-24a (Table S2). The superfolder green fluorescent protein (sfGFP) sequences were located between RBS and T7 termination on the pET-23a (Table S2). The smaller the vectors, the lighter the burden, the higher the protein synthesis [21]. Therefore, the widely used and relatively simple pET23a was chosen as the original vector in the plasmid construction. The tRNA sequences were located between ribozyme and T7 termination on the pET-23a (Table S2). The His-tag was added at the end of sfGFP and aaRS for protein purification. The target protein in this study was sfGFP, and the selected incorporation sites were 23 and 35. The dependence of modified protein yields on the site of UNAA incorporation is called the position effect, which greatly affect the yield and suppression efficiencies [19,22,23]. Chen et al. mutated the codon at site 23 in sfGFP into TAG, which proved efficient incorporation of pPaF in 23TAG-sfGFP [24]. The other selected site for multiple UNAA incorporation was at site 35, which site was reported as a permissive site for UNAAs incorporation into deGFP [18].The suppression efficiency is defined as the ratio of the modified protein yield to the natural protein yield. In the single-site incorporation experiment, the codons of the corresponding site were mutated into TAG, TAA or TGA termination codon. In the multiple-site incorporation experiment, the codons of two sites were mutated to TAA and TAG termination codons. Maps of all plasmids were provided in Figs. S1–10.

### 2.3 Cell extract preparation

The strains used as cell extracts in this study were all C321.ΔA with their genomes recoded [9]. First, single colonies were selected and inoculated in 20 mL LB medium and incubated overnight at 30 °C (200 rpm). Then 12.5 mL overnight culture was inoculated in 250 mL 2 × YTP medium (10 g/L Yeast extract, 16 g/L Tryptone, 5 g/L NaCl, 40 mM K_2_HPO4, 22 mM KH_2_PO4) shaking at 30 °C (200 rpm). When the OD_600_ value was 0.6–0.8, the culture was diluted (1:20) and added into a bottle containing 1 L 2 × YTP medium shaking at 30 °C (200 rpm). Monitored the growth status during the cultivation. In the middle and late stages of the logarithmic growth phase (about 5–6 h), centrifuged at 10,000×*g* for 10 min to collect cells. The bacteria were washed with S30A buffer (14 mM Mg-glutamate, 60 mM K-glutamate, 50 mM Tris, pH 7.7) twice, and the bacteria were considered wet weight. 1 mL S30A was added to 1 g of bacteria, and the bacteria were suspended. The bacteria were broken twice with a high-pressure breaker (15,000 Pa). Then broken samples were centrifuged at 4 °C for 30 min (13,000×*g*), 3 μl of 1 M DTT were added to each 1 mL supernatant, and samples were incubated in the dark at 37 °C for 80 min (120 rpm). After dialysis in S30B dialysis buffer (14 mM Mg-glutamate, 60 mM K-glutamate, 5 mM Tris, pH 8.2) at 4 °C overnight, the samples were centrifuged, frozen and stored in the refrigerator at −80 °C [25].

### 2.4 Preparation of tRNAs

The target fragment and vector of tRNA were amplified and recovered separately by PCR. The Gibson assembly method was used to connect the target fragment and the vector. The ligated plasmid was chemically transformed into host cell DH5α by the chemical transformation method.

Pfu high-fidelity DNA polymerase was used to perform PCR on tRNA. The reaction components included: 38 μl ddH_2_O, 5 μl 10 × pfu buffer, 1 μl dNTPs (10 mM), 1.75 μl template, 2 μl forward primer, 2 μl reverse primer and 0.25 μl pfu polymerase. The program was run at 94 °C for 3 min, followed by 35 cycles of 94 °C for 30 s, 57 °C for 30 s and 72 °C for 2 min/kb. The final extension was running at 72 °C for 10 min and 4 °C forever. After PCR, the bands were confirmed by DNA agarose gel electrophoresis. The entire PCR product was then recovered, and DNA was recovered using ethanol precipitation. The specific method was to add 1/10 volume of sodium acetate and 1/3 of absolute ethanol to the product, and placed it at −20 °C overnight. Collected the pellet after centrifugation, washed the pellet twice with 70% ethanol, dried the pellet, and added an appropriate amount of water to dissolve the pellet and test the concentration [24].

### 2.5 Preparation of aaRSs

The host cell for aaRS expression was BL21(DE3). First, single colonies were selected and inoculated in 10 mL LB liquid medium, at 37 °C, 220 rpm, overnight cultured. The culture was expanded at 5% of the inoculum. At an OD600 of 0.6–0.8, 1000 μl 1 M isopropyl β-d-1-thiogalactopyranoside (IPTG) was added to a final concentration of 1 mM. After 9–12 h incubation at 30 °C and 220 rpm, the cultures were pelleted and washed twice with 20 ml lysis buffer (20 mM Na_2_HPO_4._12H_2_O, 50 mM NaCl, 30 mM Imidazole, H_3_PO_4_, PH7.4). The lysis buffer was added to resuspend the bacteria, so that the final OD600 value of the bacteria after dilution was 40–60. A high-pressure disruptor was used to repeatedly disrupt the bacteria twice. The lysate was clarified by centrifugation at 4 °C at 12000 rpm for at least 30 min. Following filtration with 0.45 μm water filters, cell lysate was purified by 5 mL EzFast Ni HP column using ÄKTA Prime system, and then was dialyzed against sterile 1 × PBS buffer (pH 7.4) overnight as previously. Then the protein concentrations were determined by using Quick Start Bradford Protein Assay Kit. When necessary, the proteins were concentrated using Amicon Ultra centrifugal device (10 kDa). Finally, 20% (v/v%) sucrose was added to the protein solution, and stored at −80 °C [ [26]].

### 2.6 Cell-free unnatural protein synthesis reactions

CFUPS reaction was performed in a 1.5-mL Eppendorf tube at 30 °C with a final volume of 20 μl as described previously [25,26]. 20 μl reaction solutions contained, unless otherwise noted: 175 mM potassium glutamate, 10 mM ammonium glutamate, 2.7 mM potassium oxalate, 20 mM magnesium glutamate, 0.1 mM PEP, 0.2 μl of T7 RNA polymerase [27], 50 mM 19 amino acids, 25 × NTP Mix (1 mM putrescine, 1.5 mM spermidine, 0.33 mM NAD, 1.2 mM ATP, 0.86 mM CTP, 0.86 mM GTP, 0.86 mM UTP, 0.27 mM CoA, and 170 g/mL tRNA), 5 μl of cell extract, 300 ng DNA template, 0.3 mM aaRS, 1200 ng tRNA, 2 mM UNAA, and ddH_2_O. The reactions were incubated at 30 °C for 12 h, unless stated otherwise.

### 2.7 Detection of expression products

First, the sfGFP fluorescence was measured using a fluorescence plate reader, ARVO SX (PerkinElmer, Waltham, MA, USA), with excitation at 485 nm and emission at 535 nm. The quantification method of unnatural proteins was as described [28]. Secondly, it was necessary to detect whether UNAA was incorporated in 23/35 site TAG/TAA/TGA-sfGFP by mass spectrometry. Finally, it was necessary to detect whether two UNAAs were incorporated in 23TAA35TAG-sfGFP by mass spectrometry. Prior to mass spectrometry, the product was purified using affinity chromatography.

### 2.8 Mass spectrometric detection and analysis

One mL of unnatural sfGFP with a C-terminal His-tag was expressed in a 16-well plate, purified using His-tag affinity chromatography, and then concentrated by a Amicon Ultra centrifugal device (10 kDa). Following 10% SDS-PAGE analysis with staining with Coomassie blue dye, the target protein band was cut from the gel and sent to the protein analysis platform at Tsinghua University for mass spectrometry detection (AB Sciex 4800 plus TOF/TOF). Protein Prospector were used to analyze the mass spectrum results (https://prospector2.ucsf.edu/prospector/mshome.htm). The software could analyze the reliability of the protein, and provide information such as the coverage rate of amino acid sequence, the number of peptide segments, the abundance of protein, physicochemical properties, and so on.

### 2.9 Variance analysis of the orthogonal test

After obtaining the experimental data, the sum of test indexes of the same level of each factor and the sum of test indexes of all test numbers were calculated. The average of the same level test index of each factor was also calculated. Then the sum of square and degree freedom of each factor and error variation were calculated to obtain the mean square. Then F-test was used to analyze the results to evaluate the significance of the factors [24].

## 3 Results and discussion

### 3.1 Design of expression templates for single UNAA incorporation

**Fig. 2:**
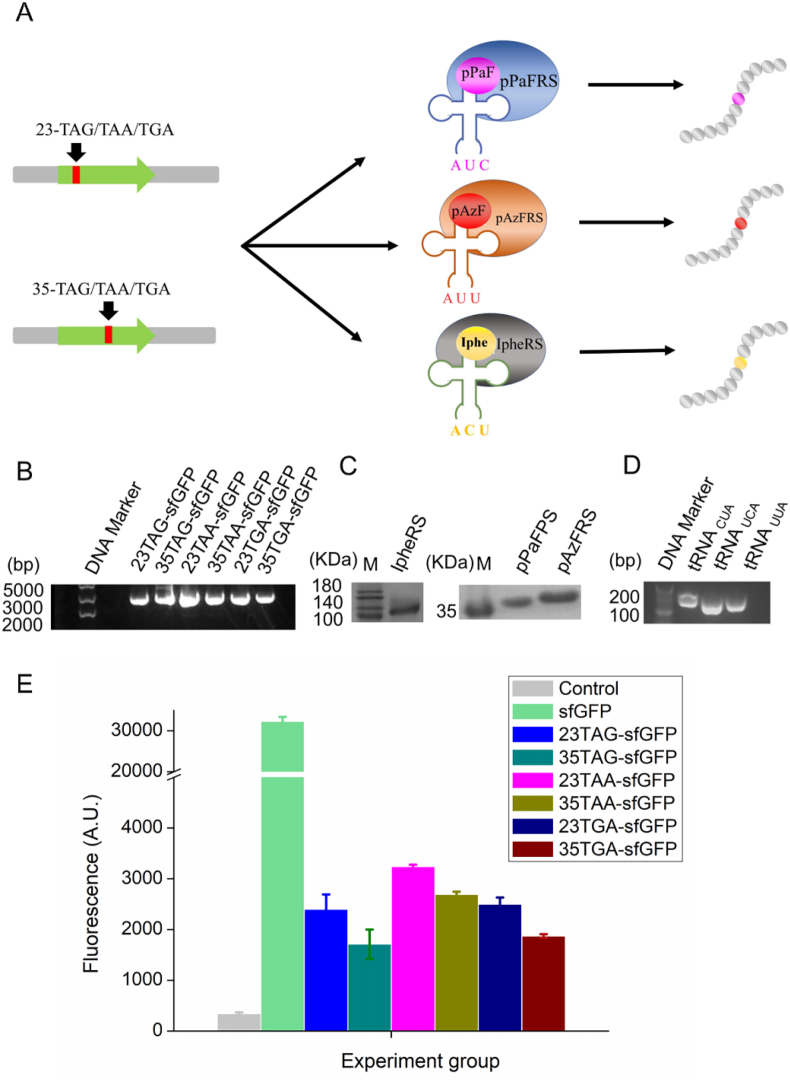
The UNAA incorporation at single site in sfGFP based on CFUPS. (A) Design of study. (B) Electrophoresis results of target protein plasmids. From left to right were 23TAG-sfGFP, 35TAG-sfGFP, 23TAA-sfGFP, 35TAA-sfGFP, 23TGA-sfGFP, 35TGA-sfGFP. The size of the mutant plasmid was 4346 bp, and the agarose gel electrophoresis was performed with 1% DNA gel. The plasmid gene was the only product. (C) SDS-PAGE results of purified aaRSs. The molecular weights of IpheRS, pPaFRS, and pAzFRS were 88.2 kDa, 36.0 kDa, and 36.1 kDa. (D) Electrophoresis results of tRNA gene templates. The sizes of the three tRNA fragments were tRNA_CUA_ (156 bp), tRNA_UCA_ (155 bp), and tRNA_UUA_ (156 bp). (E) The sfGFP expression with or without the UNAA incorporation. The CFUPS system included 0.5 mM aaRS, 75 μg/mL tRNA, and 10 mM UNAA. The reactions were incubated at 30 °C for 12 h. The mean and standard deviations were shown (N = 3). The result indicated six sfGFP variants were expressed successfully.

Proper preparation of DNA plasmids, tRNAs and aaRSs are the first step for successful incorporation of UNAAs. In the CFUPS reactions, pPaF, tRNA_CUA_ and pPaFRS were used for TAG suppression, pAzF, tRNA_UUA_ and pAzFRS were used for TAA suppression, and Iphe, tRNA_UCA_ and IpheRS were used for TGA suppression (Fig. 2A). By verification, six DNA plasmids, three tRNA genes and three aaRSs were successfully prepared **(**Fig. 2B–D**)**. Thus, these products were favorable for subsequent CFUPS reactions. By CFUPS reaction as described [25], six modified sfGFP proteins were proved to be expressed successfully (Fig. 2E).

In order to improve the expression of unnatural protein and the incorporation efficiency of UNAA, the reagent components of cell-free system needed to be further optimized, including the redox environment, Mg^2+^ and orthogonal translation system (OTS) components (Fig. S11). Optimizing the redox environment could improve the catalytic performance of the system. Mg^2+^ could affect the interactions between proteins and nucleic acids in biological processes such as protein synthesis. Furthermore, adding the detached OTS components into the CFUPS reaction was proved a feasible way for flexible OTS operation. Optimizing OTS component concentration has developed to be a highly efficient and adaptive way of OTS orthogonality enhancement. Therefore, in the next section, optimizing the cell-free system components was necessary. It is hypothesized that by modifying the *E. coli* CFPS system components of known protocol and conducting new experiments on different systems, the optimal system components suitable for CFPS system were obtained [21].

### 3.2 Exploration of the optimal redox environment for single UNAA incorporation

**Fig. 3:**
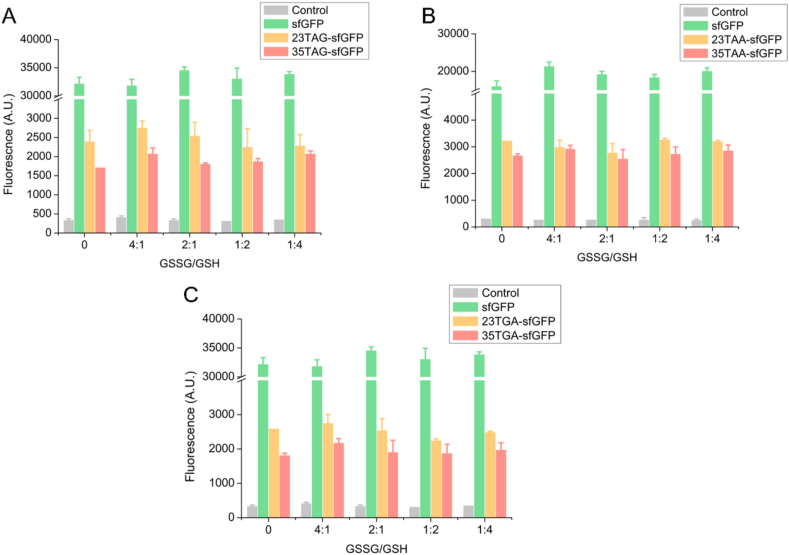
Effects of the redox environment on protein expression for termination codon suppression based on CFUPS system. The cell-free system was incubated under different redox conditions for 12 h at 30 °C, in which the GSSG to GSH ratio increased from 4:1 to 1:4. **(A**–**C)** Termination codon suppression for TAG, TAA and TGA, respectively. The mean and standard deviations were shown (N = 3).

The redox environment in the system is a key factor for the protein synthesis, because it affects not only the catalytic performance of the system but also affect the conformation of functional proteins [29]. The redox environment can be regulated by the oxidized and reduced glutathione (GSSG and GSH). The effects of different redox environments on protein expression in cell-free system were explored with different ratios of GSSG to GSH. As shown in Fig. 3, It was found that the adjustment of the redox environment had little effect on the wild-type or unnatural sfGFP protein expression in the CFUPS system. Therefore, the effects of the redox environment on CFUPS would no longer be considered in further studies.

### 3.3 Optimization of Mg^2+^ level for single UNAA incorporation

**Fig. 4:**
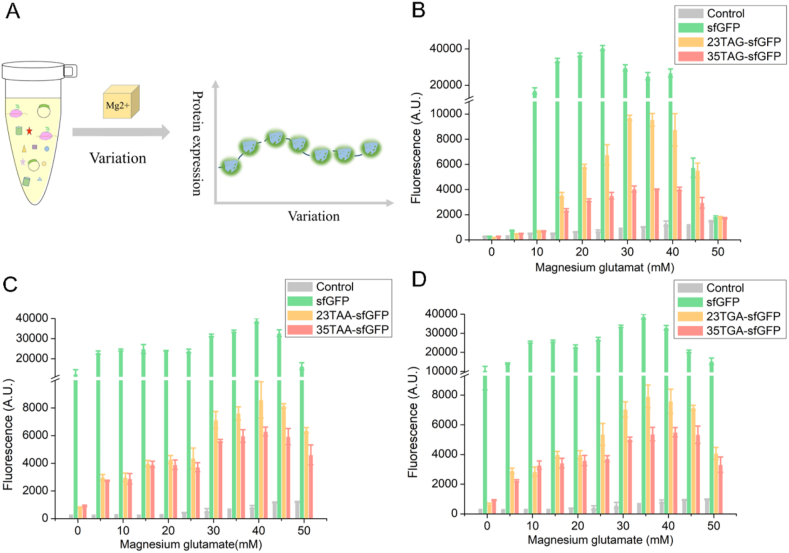
The effects of Mg^2+^ on CFUPS. Mg^2+^ at different concentrations (0–50 mM) were added to the CFUPS system, and the protein expression levels were explored. **(A)** The diagram showing the effect of Mg^2+^ on the protein expression. **(B)** The screening results of optimal Mg^2+^ concentration in TAG suppression. When Mg^2+^ was 30 mM, the highest expression of 23TAG-sfGFP and 35TAG-sfGFP was 2.6 mg/mL and 2.49 mg/mL, respectively. **(C)** The screening results of optimal Mg^2+^ concentration in TAA suppression. When Mg^2+^ was 40 mM, the highest expression of 23TAA-sfGFP and 35TAA-sfGFP was 2.58 mg/mL and 2.53 mg/mL, respectively. **(D)** The screening results of optimal Mg^2+^ concentration in TGA suppression. When Mg^2+^ was 35 mM, the highest expression of 23TGA-sfGFP and 35TGA-sfGFP was 2.56 mg/mL and 2.51 mg/mL, respectively. The mean and standard deviations were shown (N = 3).

Mg^2+^ can play an essential role in both folding and catalysis for regulating RNA and protein synthesis [30]. In the CFUPS system, Mg^2+^ not only participates in transcription and translation but also acts as activators of enzymes such as RNA polymerase and aminoacyl-tRNA synthase [21]. However, excessive Mg^2+^ leads to premature termination of protein synthesis. Therefore, in this part, the effects of Mg^2+^ ranging from 0 mM to 50 mM (Fig. 4) were explored on the protein expression in the CFUPS platform. The protein expression level increased with the addition of Mg^2+^, which indicated that adding the optimum concentration of Mg^2+^ in cell-free system could significantly improve the protein expression. The expression levels of unnatural proteins presented the same trend. With the increase of Mg^2+^ concentration, the protein expression first gradually increased, and after reaching the highest point, the expression began to decrease. When the expression of unnatural protein reached the highest point, the corresponding optimal concentrations of magnesium glutamate for TAG suppression, TAA suppression, and TGA suppression were 30 mM, 40 mM and 35 mM, respectively, which further showed that Mg^2+^ could significantly increase the expression of proteins in the CFUPS system.

### 3.4 Optimization of tRNA level for single UNAA incorporation

**Fig. 5:**
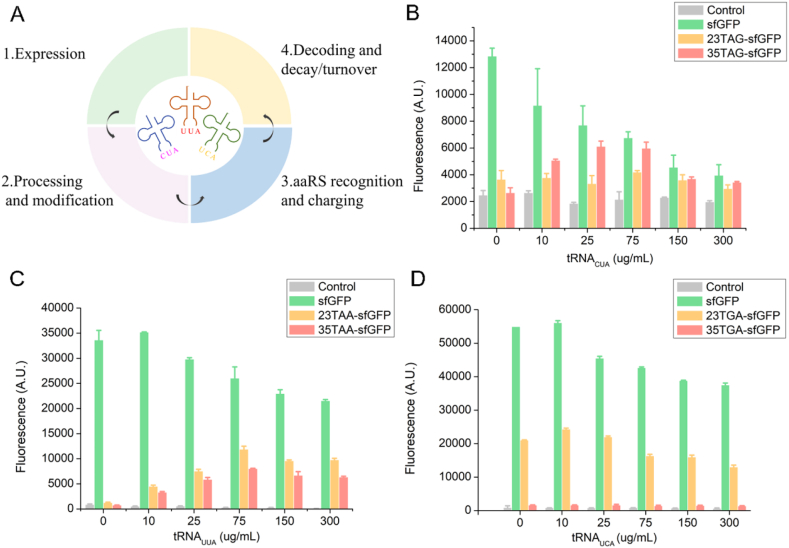
Optimal tRNA concentration screening. Three tRNAs at different concentrations (0–300 μg/mL) were added to the CFUPS system, and the protein expression levels were explored. **(A)** Orthogonal tRNAs participate in all stages of translation. **(B)** Screening of optimal concentration of tRNA_CUA_ in the TAG suppression. When tRNA_CUA_ was 75 μg/mL, the highest expression of 23TAG-sfGFP and 35TAG-sfGFP was 2.49 mg/mL and 2.52 mg/mL, respectively. **(C)** Screening of optimal concentration of tRNA_UUA_ in the TAA suppression. When tRNA_UUA_ was 75 μg/mL, the highest expression of 23TAA-sfGFP and 35TAA-sfGFP was 2.64 mg/mL and 2.57 mg/mL, respectively. **(D)** Screening of optimal concentration of tRNA_UCA_ in the TGA suppression. When tRNA_UCA_ was 10 μg/mL, the highest expression of 23TGA-sfGFP and 35TGA-sfGFP was 2.89 mg/mL and 2.44 mg/mL, respectively. The mean and standard deviations were shown (N = 3).

In the CFUPS system, tRNA can directly affect the carrying efficiency of UNAA and the expression level of unnatural proteins [31]. Therefore, the effects of tRNAs at different concentrations ranging from 0 μg/mL to 300 μg/mL on the protein expression were explored in the CFUPS platform, as shown in Fig. 5. It was observed that adding tRNA with optimal concentration could make the protein synthesis reach the highest level. For natural sfGFP protein, with the increasing tRNA levels in the CFUPS system, its expression gradually decreased. This trend indicated that the addition of exogenous tRNA had a toxic effect on the CFUPS system and inhibited the expression of the protein. However, the expression level of unnatural protein showed an upward trend at first. When it reached the top, the unnatural protein expression began to decrease. When the expression of sfGFP variants reached the highest point, the corresponding tRNA levels for TAG suppression, TAA suppression, and TGA suppression were 75 μg/mL, 75 μg/mL, and 10 μg/mL, respectively.

### 3.5 Optimization of aaRS level for single UNAA incorporation

**Fig. 6:**
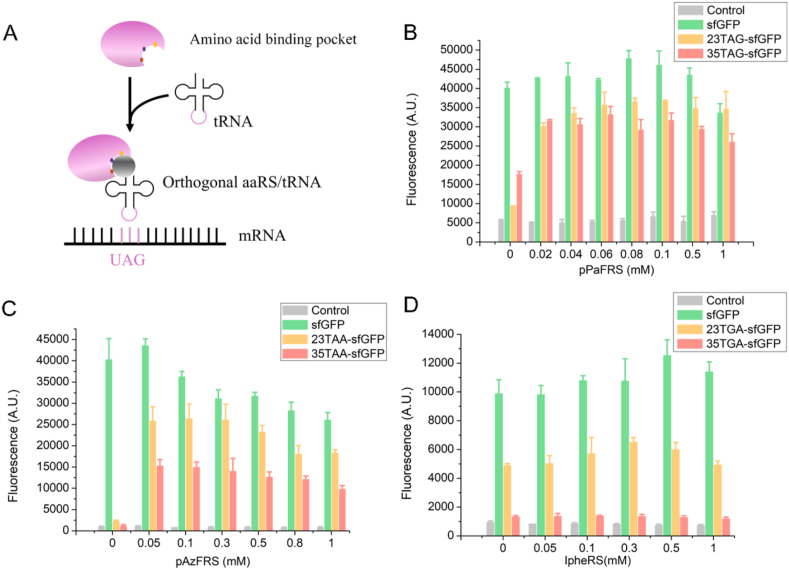
Optimal aaRS concentration screening. Three aaRSs at different concentrations (0–1 mM) were tested in the CFUPS system. **(A)** Orthogonal aaRS function in the translation. The orthogonal aaRS was introduced to charge an orthogonal tRNA with a UNAA, enabling site-specific targeted incorporation. **(B)** Screening the optimal concentration of pPaFRS in TAG suppression. When pPaFRS was 0.06 mM, the highest expression of 23TAG-sfGFP and 35TAG-sfGFP was 3.12 mg/mL and 3.07 mg/mL, respectively. **(C)** Screening the optimal concentration of pAzFRS in TAA suppression. When pAzFRS was 0.1 mM, the highest expression of 23TAA-sfGFP and 35TAA-sfGFP was 2.94 mg/mL and 2.7 mg/mL, respectively. **(D)** Screening the optimal concentration of IPheRS in TGA suppression. When IpheRS was 0.3 mM, the highest expression of 23TGA-sfGFP and 35TGA-sfGFP was 2.54 mg/mL and 2.4 mg/mL, respectively. The mean and standard deviations were shown (N = 3).

Aminoacyl-tRNA synthetase is involved in aminoacylating tRNAs, which serves as amino acid donors for biosynthetic processes, and it is also essential to RNA splicing, transcriptional regulation, translation, and other aspects of life homeostasis [32]. To optimize the expression of unnatural proteins, it was necessary to adjust the aaRS level in the CFUPS system. The aaRS concentrations ranging from 0 mM to 1 mM were explored, as shown in Fig. 6(B-D). With the increase of aaRS levels, the unnatural protein expression showed a trend of increasing first. When the concentrations of aaRSs added in the CFUPS system for TAG suppression, TAA suppression, and TGA suppression were 0.06 mM, 0.1 mM, and 0.3 mM, respectively, the protein expression level reached the highest. However, too many aaRSs were detrimental to the protein expression, which might increase the burden of the CFUPS system.

### 3.6 Optimization of UNAA level for single UNAA incorporation

**Fig. 7:**
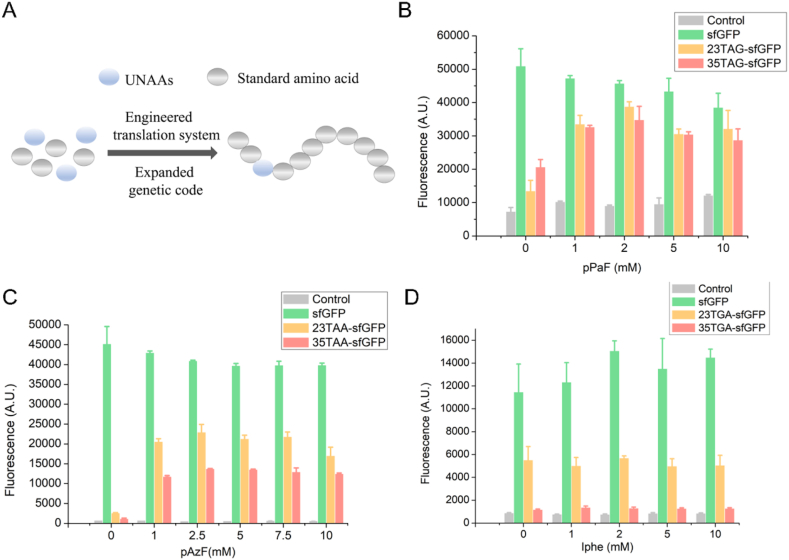
Optimal UNAA concentration screening. The UNAAs were added to the cell-free system, incubating for 12 h at 30 °C. **(A)** The diagram showing the incorporation of UNAA. **(B)** Screening the optimal concentration of pPaF in TAG suppression. When pPaF was 2 mM, the highest expression of 23TAG-sfGFP and 35TAG-sfGFP was 3.18 mg/mL and 3.1 mg/mL, respectively. **(C)** Screening the optimal concentration of pAzF in TAA suppression. When pAzF was 2.5 mM, the highest expression of 23TAA-sfGFP and 35TAA-sfGFP was 2.87 mg/mL and 2.7 mg/mL, respectively. **(D)** Screening the optimal concentration of Iphe in TGA suppression. The mean and standard deviations were shown (N = 3).

Moreover, to avoid the accumulation of truncated protein products and further increase the incorporation efficiency, the UNAA levels from 0 mM to 10 mM were evaluated. As shown in Fig. 7, the excess addition of UNAAs could inhibit the natural protein synthesis. When the UNAA concentrations for TAG suppression and TAA suppression were 2 mM and 2.5 mM, respectively, the unnatural protein expression reached the highest. However, for TGA suppression, the increasing of Iphe concentrations from 0 to 10 mM could not improve the protein expression. It might be attributed to that, Iphe could be recognized by natural endogenous tRNAs and aaRSs. Hence the incorporation of Iphe would not be explored in further studies.

### 3.7 Mass spectroscopy analysis of the modified protein

**Fig. 8:**
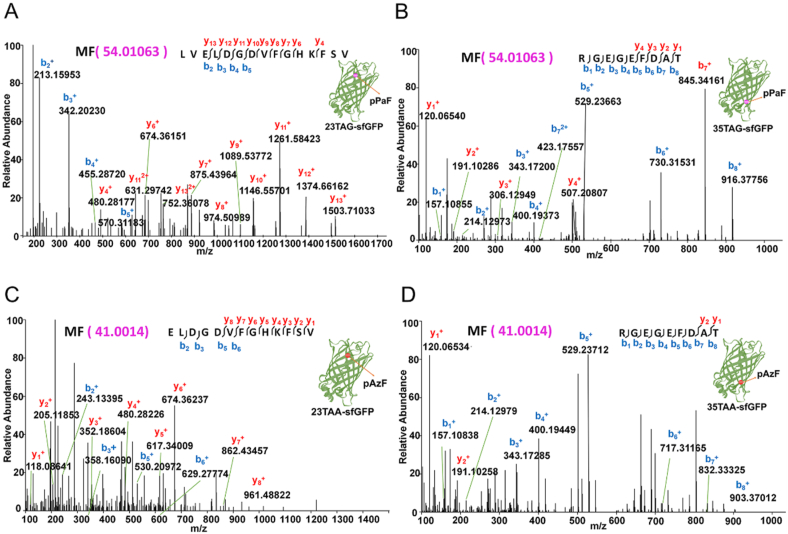
Mass spectrometry analysis for confirming the UNAA incorporation. Fragmentation of each peptide is predicted to yield a series of b ions (blue) and a series of y ions (red). Ion peaks were assigned manually, along with precursor ion masses. **(A)** MS spectra confirming pPaF incorporation at site 23. **(B)** MS spectra confirming pPaF incorporation at site 35. **(C)** MS spectra confirming pAzF incorporation at site 23. **(D)** MS spectra confirming pAzF incorporation at site 35. (For interpretation of the references to colour in this figure legend, the reader is referred to the Web version of this article.)

Furthermore, the mass spectroscopy analysis was performed to confirm the actual incorporation of UNAAs. The molecular weight of pPaF is 54.01063 g/mol, and the LC-MS/MS analysis confirmed that pPaF had been successfully incorporated into the sfGFP, in either site 23 or site 35 (Fig. 8A–B). The molecular weight of pAzF is 41.0014 g/mol, and the LC-MS/MS analysis confirmed that pAzF had been successfully incorporated into the sfGFP, in either site 23 or site 35 (Fig. 8C–D). However, the LC-MS/MS analysis could not detect the incorporation of Iphe in sfGFP, which further confirmed the poor orthogonality of Iphe, tRNA_UCA_, and IpheRS tested in this study. Furthermore, in terms of the frequency of use of termination codons, TAG termination codon was the most widely used, TAA termination codon was the second. However, TGA termination codon was less used, and perhaps it is due to OTS having poor orthogonality in TGA termination codon suppression, so it was difficult to incorporate UNAA into the target protein. Therefore, only the TAG and TAA suppression methods would be performed in the subsequent multiple-site UNAAs incorporation experiments.

### 3.8 Design of expression templates for two-UNAAs incorporation

**Fig. 9:**
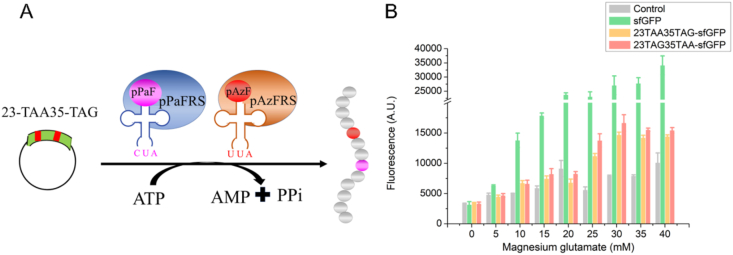
Two-UNAAs incorporation in the CFUPS system. **(A)** Design of two-UNAAs incorporation study. **(B)** Optimal Mg^2+^ concentration screening. Mg^2+^ at different concentrations (0–40 mM) were added to the CFPS system. When Mg^2+^ was 30 mM, the highest expression of 23TAA35TAG-sfGFP and 23TAG35TAA-sfGFP was 2.7 mg/mL and 2.74 mg/mL, respectively. The mean and standard deviations were shown (N = 3).

To achieve the multiple-site UNAA incorporation of different kinds of UNAAs, a combination of two different suppression methods was used, including TAG termination codon suppression method and TAA termination codon suppression method. A target protein 23TAA35TAG-sfGFP was established (Fig. 9A). The plasmid map was provided in Fig. S7. The agarose gel electrophoresis test proved that the plasmid was extracted successfully (Fig. S12). As described before, Mg^2+^ could significantly affect the protein expression in the CFUPS system. To increase the protein yield, the concentration of Mg^2+^ was optimized. As shown in Fig. 9B, it showed that, with the increase of Mg^2+^ concentration, the 23TAA35TAG-sfGFP expression showed a trend of first increasing and then decreasing. When the Mg^2+^ concentration was 30 mM, the expression reached the highest.

### 3.9 Optimization of OTS component concentrations

To efficiently synthesize unnatural proteins, the optimal OTS component concentrations with the lowest toxicity and the highest protein expression need to be explored. The orthogonal design of experiments was performed, as shown in Table 1. There were two OTS component sets in the experiment. Each component set contained 3 elements, and 5 concentration gradients were designed for each factor.

**Table 1:** Screening the optimal conditions for the CFUPS with the incorporation of two UNAAs.

Factor	pPaFRS (mM)	pAzFRS (mM)	tRNA_CUA_ (μg/mL)	tRNA_UUA_ (μg/mL)	pPaF (mM)	pAzF (mM)	Fluorescence (A.U.)
1	0.02	0.02	10	10	1	1	21700
2	0.02	0.05	25	25	2.5	2.5	24789
3	0.02	0.1	75	75	5	5	19500
4	0.02	0.5	150	150	7.5	7.5	19000
5	0.02	1	300	300	10	10	20789
6	0.05	0.02	25	75	7.5	10	21000
7	0.05	0.05	75	150	10	1	19200
8	0.05	0.1	150	300	1	2.5	23789
9	0.05	0.5	300	10	2.5	5	21789
10	0.05	1	10	25	5	7.5	17389
11	0.1	0.02	75	300	2.5	7.5	19000
12	0.1	0.05	150	10	5	10	20789
13	0.1	0.1	300	25	7.5	1	21500
14	0.1	0.5	10	75	10	2.5	17789
15	0.1	1	25	150	1	5	14789
16	0.5	0.02	150	25	10	5	20789
17	0.5	0.05	300	75	1	7.5	18789
18	0.5	0.1	10	150	2.5	10	19789
19	0.5	0.5	25	300	5	1	19970
20	0.5	1	75	10	7.5	5	21789
21	1	0.02	300	150	5	2.5	16789
22	1	0.05	10	300	7.5	5	17090
23	1	0.1	25	10	10	7.5	22500
24	1	0.5	75	25	1	10	18540
25	1	1	150	75	2.5	1	17560

**Fig. 10:**
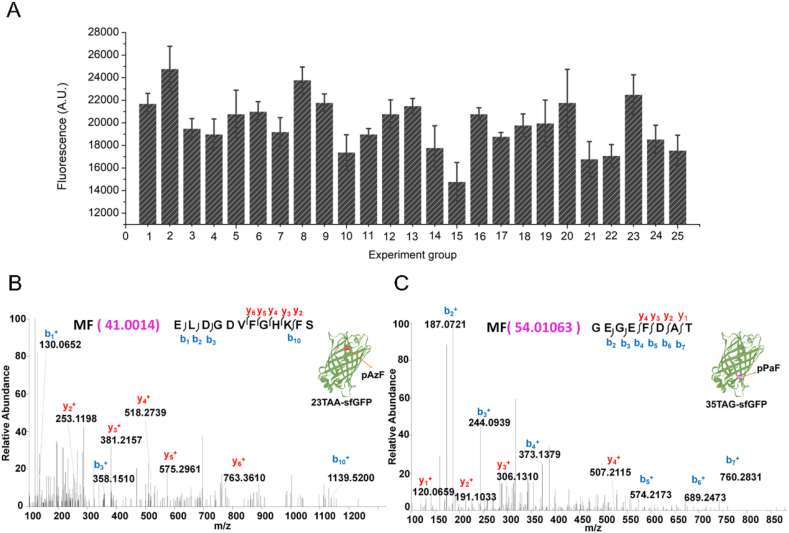
Two UNAAs incorporations in the CFUPS system. **(A)** Optimal OTS components concentration screening. When the OTS components of group 2 were added to the cell-free system, the highest expression of 23TAG35TAA-sfGFP was 2.9 mg/mL. The mean and standard deviations were shown (N = 3). **(B)** MS spectra confirming pAzF incorporation at site 23. **(C)** MS spectra confirming pPaF incorporation at site 35. According to the secondary mass spectrogram, blue was the matched b ion, and red was the matched y ion. The precursor ions confirmed the incorporation of the two UNAAs. (For interpretation of the references to colour in this figure legend, the reader is referred to the Web version of this article.)

As shown in Fig. 10A, after 25 groups of OTS components were tested, the group of experiment 2 showed the highest protein expression. Therefore, the optimal OTS concentrations were 0.02 mM pPaFRS, 25 μg/mL tRNA_CUA_, 2.5 mM pPaF, 0.05 mM pAzFRS, 25 μg/mL tRNA_UUA_, and 2.5 mM pAzF, which would be used in the subsequent protein expression and mass spectrometry verification. In addition, analysis of variance was used to explore the key factors affecting the expression of the system (Table 2). Sum of square (SS), degree freedom (*df*), mean square (MS) and F statistic (F) were displayed. F (0.05) was the F statistic at 95% confidence coefficient. In this experiment, since each group had the same *df*, F statistic represented the disturbance of each factor to the system. The larger F statistic was, the larger the disturbance was. The results of F-test indicated that the concentration of pAzFRS (F = 17.535, 4 and 25 *df*) and pAzF (F = 13.56, 4 and 25 *df*) had a significant impact on the incorporation efficiency of the cell-free system. The concentration of pPaFRS (F = 2.979, 4 and 25 *df*), tRNA_CUA_ (F = 2.868, 4 and 25 *df*), tRNA_UUA_ (F = 2.429, 4 and 25 *df*), and pPaF (F = 2.963, 4 and 25 *df*) was not significant. Among 25 tested groups, the concentration of pAzFRS had the largest effect on the CFUPS system. These experiment design and findings also indicated that the method established in this study could quickly and easily screen out the optimal OTS components.

**Table 2:** Analysis of variance for exploring the key factors affecting the expression of the system.

	SS	df	MS	F	F（0.05）
pPaFRS	9.48E+06	4	2.37E+06	2.979	0.039
pAzFRS	5.58E+07	4	1.39E+07	17.535	0
tRNA_CUA_	9.13E+06	4	2.28E+06	2.868	0.044
tRNA_UUA_	7.73E+06	4	1.93E+06	2.429	0.074
pPaF	9.43E+06	4	2.36E+06	2.963	0.039
pAzF	4.31E+07	4	1.08E+07	13.56	0
Error	1.99E+07	25	7.95E+05		

In this table, “SS” meant sum of square. “df” meant degree freedom. “MS” meant mean square. “F” meant F statistic. F (0.05) was the F statistic at 95% confidence coefficient.

To confirm whether two UNAAs were successfully incorporated in the single target protein, the purified unnatural protein from the group of Experiment 2 was analyzed by the mass spectroscopy. The molecular weight of pPaF is 54.01063 g/mol and the molecular weight of pAzF is 41.0014 g/mol. As shown in Fig. 10B–C, the LC-MS/MS analysis confirmed that pPaF and pAzF had been successfully incorporated into the sfGFP protein.

## 4 Conclusion

In this study, the CFUPS system for incorporating two distinct UNAAs into a single protein was established. After exploring the single UNAA incorporation efficiency in three different termination codon suppression methods, two termination codons TAG and TAA were employed for two-UNAA incorporation. Accordingly, two sets of OTSs were used, including the pair of pPaFRS, tRNA_CUA_, and pPaF, and the pair of pAzFRS, tRNA_UUA_, and pAzF. Different reaction environments or conditions were investigated for improved unnatural protein yields. In the future work, the CFUPS system developed in this study could be used for the synthesis of functional unnatural proteins, such as enzymes and protein materials. It will continue to incubate emerging applications in biopharmaceuticals, mirror proteins, molecular labeling, and biomaterials. For the termination codon suppression method, evolving ribosomes could be used to improve the UNAAs incorporation by improving the orthogonality of engineered orthogonal ribosomal translational systems. Toward the incorporation of more distinct UNAAS into a single protein, other UNAA incorporation approached need to be developed and combined, such as code shift suppression, codon redistribution, and unnatural base pair.

## Ethics approval

This article does not contain any studies with human participants or experimental animals performed by any of the authors.

## CRediT authorship contribution statement

**Jiaqi Hou:** Investigation, Writing - original draft preparation. **Xinjie Chen:** Investigation, Writing - original draft preparation. **Nan Jiang:** Investigation, Writing - original draft preparation. **Yanan Wang:** Data Curation. **Yi Cui:** Data Curation. **Lianju Ma:** Supervision. **Ying Lin:** Supervision. **Yuan Lu:** Writing- Reviewing and Editing, Supervision, Project administration, Funding acquisition.

## Declaration of interests

The authors declare that they have no known competing financial interests or personal relationships that could have appeared to influence the work reported in this paper.
