# Supplementary material for: Toward efficient multiple-site incorporation of unnatural amino acids using cell-free translation system
Source: Synth Syst Biotechnol. 2021 Dec 23;7(1):522–32. doi: 10.1016/j.synbio.2021.12.007 (PMC8718814; doi:10.1016/j.synbio.2021.12.007)
Supplement: Multimedia component 1 [file mmc1.docx]

**Supporting Information**

**Toward efficient multiple-site incorporation of unnatural amino acids using cell-free translation system**

Jiaqi Hou ^1,2,#^, Nan Jiang ^1,2,#^, Xinjie Chen ^2,#^, Yanan Wang ^1,2^, Yi Cui ^1,2^, Lianju Ma ^1,^*, Ying Lin ^3,^*, Yuan Lu ^2,^*

^1^ College of Life Sciences, Shenyang Normal University, Shenyang 110034, China.

^2^ Key Laboratory of Industrial Biocatalysis, Ministry of Education, Department of Chemical Engineering, Tsinghua University, Beijing 100084, China.

^3^ Guangdong Key Laboratory of Fermentation and Enzyme Engineering, School of Biology and Biological Engineering, South China University of Technology, Guangzhou 510006, China.

# These authors contributed equally to this work.

*** Correspondence:** Lianju Ma, malianju@163.com (Lianju Ma); Ying Lin, feylin@scut.edu.cn (Ying Lin); Yuan Lu, yuanlu@tsinghua.edu.cn (Yuan Lu).

# Table S1 Primer sequences

| Name | Sequence | Target gene |
| --- | --- | --- |
| Y01F | GGATGGTGATGTCTAGGGTCATAAGTTTTCCGTGCGTGGC | sfGFP(23TAG) |
| Y01R | GACCCTAGACATCACCATCCAGTTCCACCAGAATAG |  |
| Y02F | GAGGGTGAATAGGACGCAACTAATGGTAAACTGACGCTG | sfGFP(35TAG) |
| Y02R | GTTGCGTCCTATTCACCCTCGCCACGCACG |  |
| Y03F | ATGGTGATGTCTAAGGTCATAAGTTTTCCGTGCGTGG | sfGFP(23TAA) |
| Y03R | ATGACCTTAGACATCACCATCCAGTTCCACCAG |  |
| Y04F | GCCACCTAAGGCAAGCTGACCCTGAAGTTCATCTG | sfGFP(35TAA) |
| Y04R | GTCAGCTTGCCTTAGGTGGCATCGCCCTCGCC |  |
| Y05F | TGGTGATGTCTGAGGTCATAAGTTTTCCGTGCGTGGC | sfGFP(23TGA) |
| Y05R | TATGACCTCAGACATCACCATCCAGTTCCACCAGAATAG |  |
| Y06F | TGAATGAGACGCAACTAATGGTAAACTGACGC | sfGFP(35TGA) |
| Y06R | CATTAGTTGCGTCTCATTCACCCTCGCCACGCAC |  |
| Y07F | GCTTTTAGATCTTAATACGACTCACTATAGGGAGACCGGC | tRNA_CUA_ |
| Y07R | TGGAGCCGGGGGTGGGATTTGAAC |  |
| Y08F | GCTTTTAGATCTTAATACGACTCACTATAGGGAGACCGGC | tRNA_UUA_ |
| Y08R | TGGTCCGGCGGAGGGGATTTGAAC |  |
| Y09F | GCTTTTAGATCTTAATACGACTCACTATAGGGAGACCGGC | tRNA_UCA_ |
| Y09R | CCTGGTGGTGGGGGGACTCG |  |
| Y10F | GATTCCGAGTCCCCCCACCACCAGGGAGACCACAACGGTTTCC | pET-23a |
| Y10R | ACCGACTGAGCTATCCCCCCGACGGTACCGGGTACCGTTTCG |  |

# Table S2 Sequences of DNA templates, tRNAs and aaRSs

| Name | Sequence |
| --- | --- |
| 23TAG-sfGFP/23TAA-sfGFP/23TGA-sfGFP | MAKGEELFTGVVPILVELDGDV*GHKFSVRGEGEGDATNGKLTLKFICTTGKLPVPWPTLVTTLTYGVQCFARYPDHMKQHDFFKSAMPEGYVQERTISFKDDGTYKTRAEVKFEGDTLVNRIELKGIDFKEDGNILGHKLEYNFNSHNVYITADKQKNGIKANFKIRHNVEDGSVQLADHYQQNTPIGDGPVLLPDNHYLSTQSVLSKDPNEKRDHMVLLEFVTAAGITHGMDELYKHHHHHH*  (* represents TAG/TAA/TGA mutations) |
| 35TAG-sfGFP/35TAA-sfGFP/35TGA-sfGFP | MAKGEELFTGVVPILVELDGDVNGHKFSVRGEGE*DATNGKLTLKFICTTGKLPVPWPTLVTTLTYGVQCFARYPDHMKQHDFFKSAMPEGYVQERTISFKDDGTYKTRAEVKFEGDTLVNRIELKGIDFKEDGNILGHKLEYNFNSHNVYITADKQKNGIKANFKIRHNVEDGSVQLADHYQQNTPIGDGPVLLPDNHYLSTQSVLSKDPNEKRDHMVLLEFVTAAGITHGMDELYKHHHHHH*  (* represents TAG/TAA/TGA mutations) |
| 23TAA35TAG-sfGFP | MAKGEELFTGVVPILVELDGDV*GHKFSVRGEGE*DATNGKLTLKFICTTGKLPVPWPTLVTTLTYGVQCFARYPDHMKQHDFFKSAMPEGYVQERTISFKDDGTYKTRAEVKFEGDTLVNRIELKGIDFKEDGNILGHKLEYNFNSHNVYITADKQKNGIKANFKIRHNVEDGSVQLADHYQQNTPIGDGPVLLPDNHYLSTQSVLSKDPNEKRDHMVLLEFVTAAGITHGMDELYKHHHHHH*  (* represents TAG/TAA/TGA mutations) |
| tRNA_CUA_ | GCTTTTAGATCTTAATACGACTCACTATAGGGAGACCGGCTGATGAGTCCGTGAGGACGAAACGGTACCCGGTACCGTCCCGGCGGTAGTTCAGCAGGGCAGAACGGCGGACTCTAAATCCGCATGGCAGGGGTTCAAATCCCCTCCGCCGGACCA |
| tRNA_UUA_ | GCTTTTAGATCTTAATACGACTCACTATAGGGAGACCGGCTGATGAGTCCGTGAGGACGAAACGGTACCCGGTACCGTCCCGGCGGTAGTTCAGCAGGGCAGAACGGCGGACTTTAAATCCGCATGGCAGGGGTTCAAATCCCCTCCGCCGGACCA |
| tRNA_UCA_ | GCTTTTAGATCTTAATACGACTCACTATAGGGAGACCGGCTGATGAGTCCGTGAGGACGAAACGGTACCCGGTACCGTCGGGGGGATAGCTCAGTCGGTAGAGCAGGGGATTTCAAATCCCCGTGTCCTTGGTTCGATTCCGAGTCCCCCCACCA |
| pPaFRS | MDEFEMIKRNTSEIISEEELREVLKKDEKSAAIGFEPSGKIHLGHYLQIKKMIDLQNAGFDIIILLADLHAYLNQKGELDEIRKIGDYNKKVFEAMGLKAKYVYGSPFQLDKDYTLNVYRLALKTTLKRARRSMELIAREDENPKVAEVIYPIMQVNAIHYAGVDVAVGGMEQRKIHMLARELLPKKVVCIHNPVLTGLDGEGKMSSSKGNFIAVDDSPEEIRAKIKKAYCPAGVVEGNPIMEIAKYFLEYPLTIKRPEKFGGDLTVNSYEELESLFKNKELHPMRLKNAVAEELIKILEPIRKRL |
| pAzFRS | MDEFEMIKRNTSEIISEEELREVLKKDEKSATIGFEPSGKIHLGHYLQIKKMIDLQNAGFDIIILLADLHAYLNQKGELDEIRKIGDYNKKVFEAMGLKAKYVYGSNFQLDKDYTLNVYRLALKTTLKRARRSMELIAREDENPKVAEVIYPIMQVNPLHYQGVDVAVGGMEQRKIHMLARELLPKKVVCIHNPVLTGLDGEGKMSSSKGNFIAVDDSPEEIRAKIKKAYCPAGVVEGNPIMEIAKYFLEYPLTIKRPEKFGGDLTVNSYEELESLFKNKELHPMRLKNAVAEELIKILEPIRKRL |
| IpheRS | KFSELWLREWVNPAIDSDALANQITMAGLEVDGVEPVAGSFHGVVVGEVVECAQHPNADKLRVTKVNVGGDRLLDIVCGAPNCRQGLRVAVATIGAVLPGDFKIKAAKLRGEPSEGMLCSFSELGISDDHSGIIELPADAPIGTDIREYLKLDDNTIEISVTPNRADCLGIIGVARDVAVLNQLPLVQPEIVPVGATIDDTLPITVEAPEACPRYLGRVVKGINVKAPTPLWMKEKLRRCGIRSIDAVVDVTNYVLLELGQPMHAFDKDRIEGGIVVRMAKEGETLVLLDGTEGKLNADTLVIADHNKALAMGGIFGGEHSGVNDETQNVLLECAFFSPLSITGRARRHGLHTDASHRYERGVDPALQHKAMERATRLLIDICGGEAGPVIDITNEATLPKRATITLRRSKLDRLIGHHIADEQVTDILRRLGCEVTEGKDEWQAVAPSWRFDMEIEEDLVEEVARVYGYNNIPDEPVQASLIMGTHREADLSLKRVKTLLNDKGYQEVITYSFVDPKVQQMIHPGVEALLLPSPISVEMSAMRLSLWTGLLATVVYNQNRQQNRVRIFESGLRFVPDTQAPLGIRQDLMLAGVICGNRYEEHWNLAKETVDFYDLKGDLESVLDLTGKLNEVEFRAEANPALHPGQSAAIYLKGERIGFVGVVHPELERKLDLNGRTLVFELEWNKLADRVVPQAREISRFPANRRDIAVVVAENVPAADILSECKKVGVNQVVGVNLFDVYRGKGVAEGYKSLAISLILQDTSRTLEEEEIAATVAKCVEALKERFQASLRD |


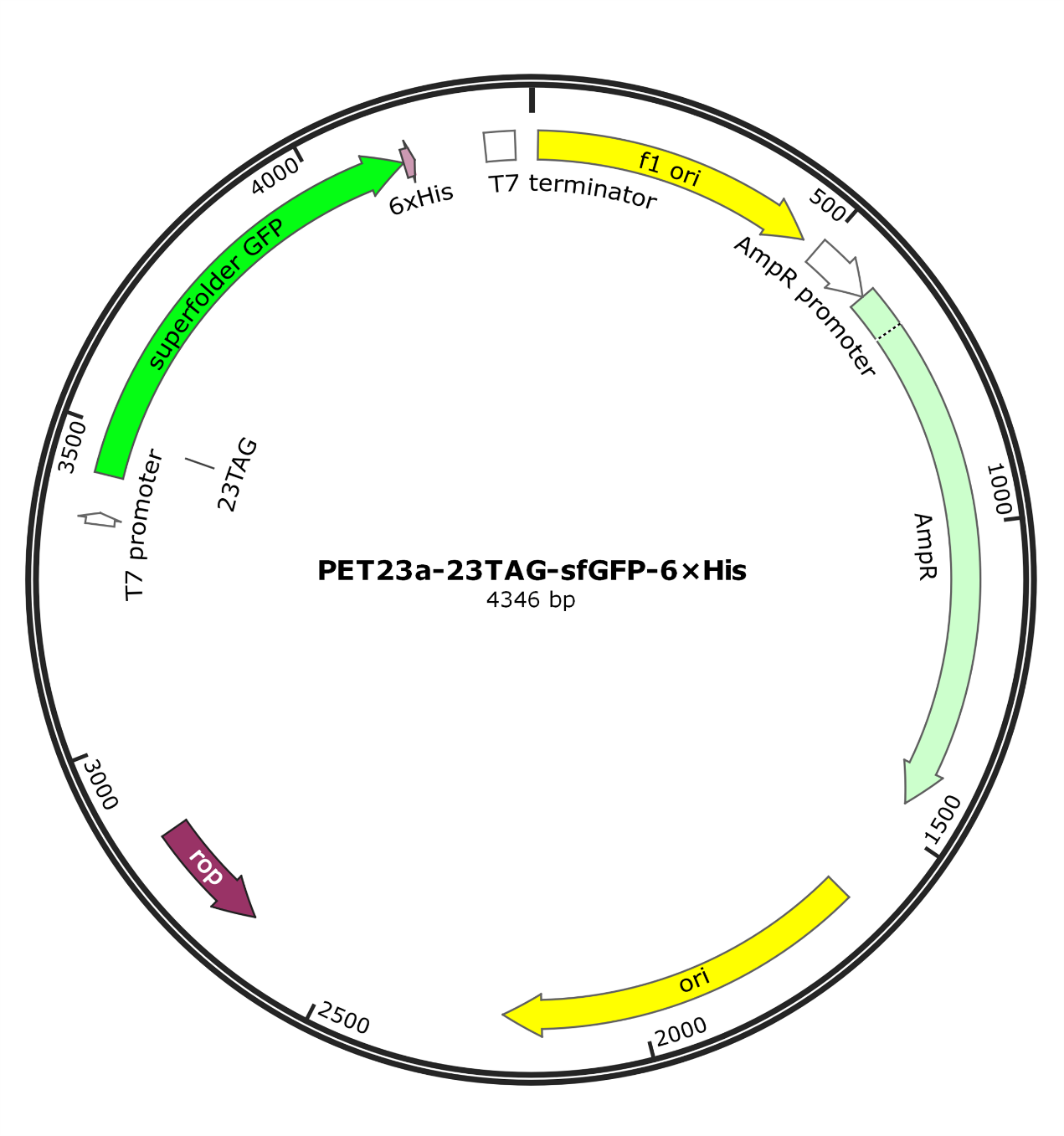


**Fig. S1** The plasmid map of pET23a-23TAG-sfGFP. The 23TAG-sfGFP sequence was located between RBS and T7 terminator on the pET-23a. The codon of 23 site in sfGFP was mutated into TAG. His-tag was at the C-terminus.


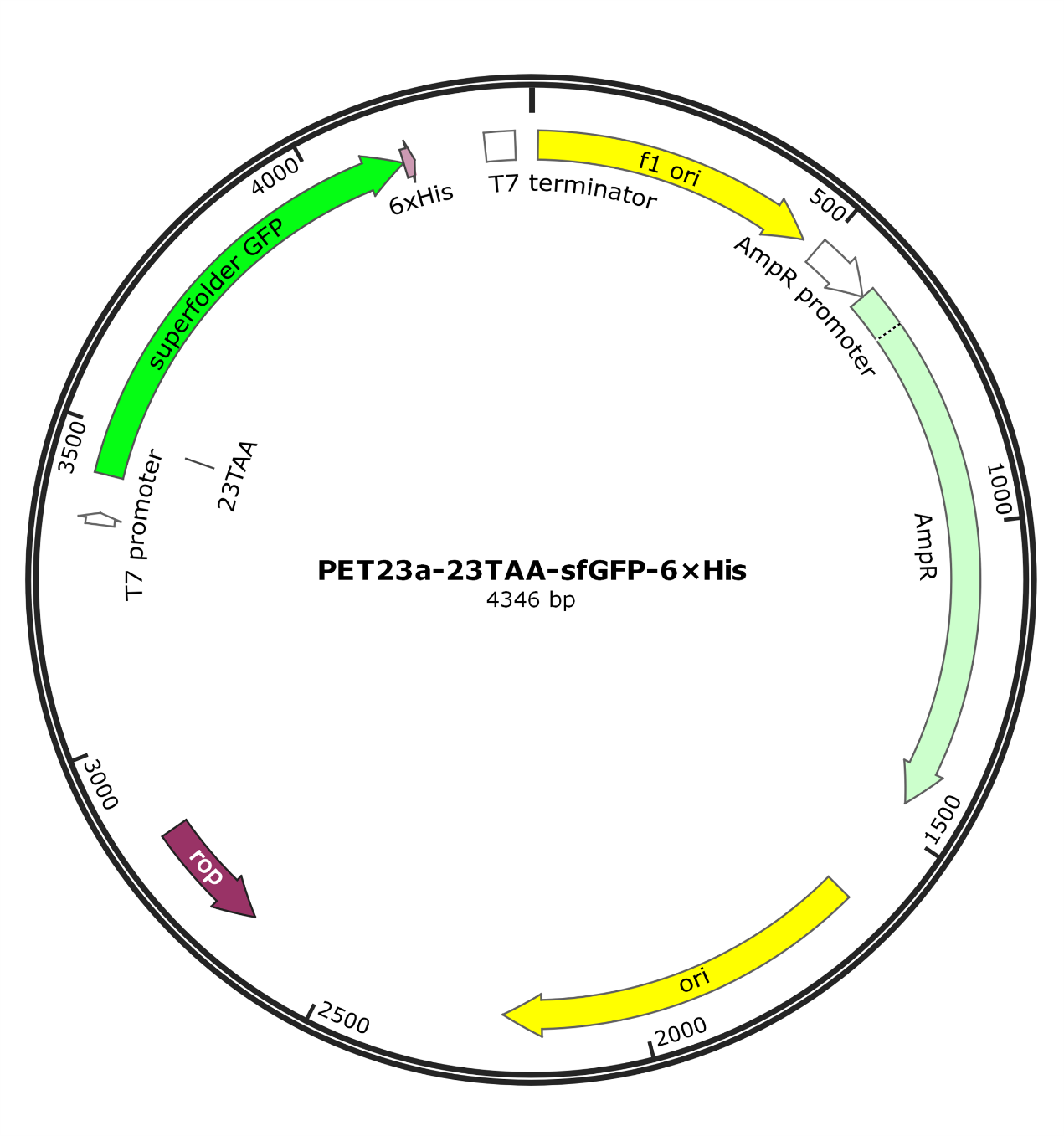


**Fig. S2** The plasmid map of pET23a-23TAA-sfGFP. The 23TAA-sfGFP sequence was located between RBS and T7 terminator on the pET-23a. The codon of 23 site in sfGFP was mutated into TAA. His-tag was at the C-terminus.


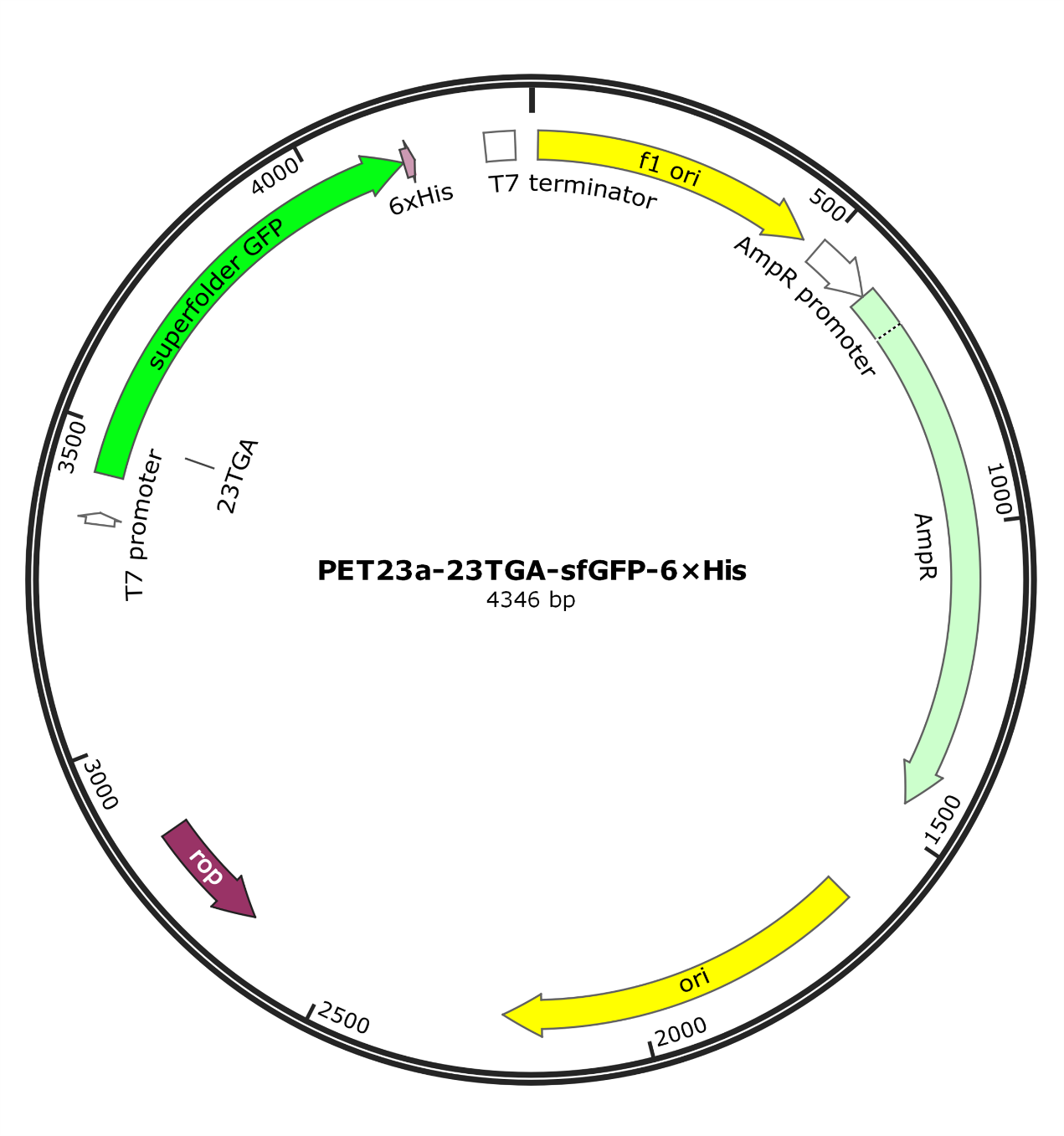


**Fig. S3** The plasmid map of pET23a-23TGA-sfGFP. The 23TGA-sfGFP sequence was located between RBS and T7 terminator on the pET-23a. The codon of 23 site in sfGFP was mutated into TGA. His-tag was at the C-terminus.


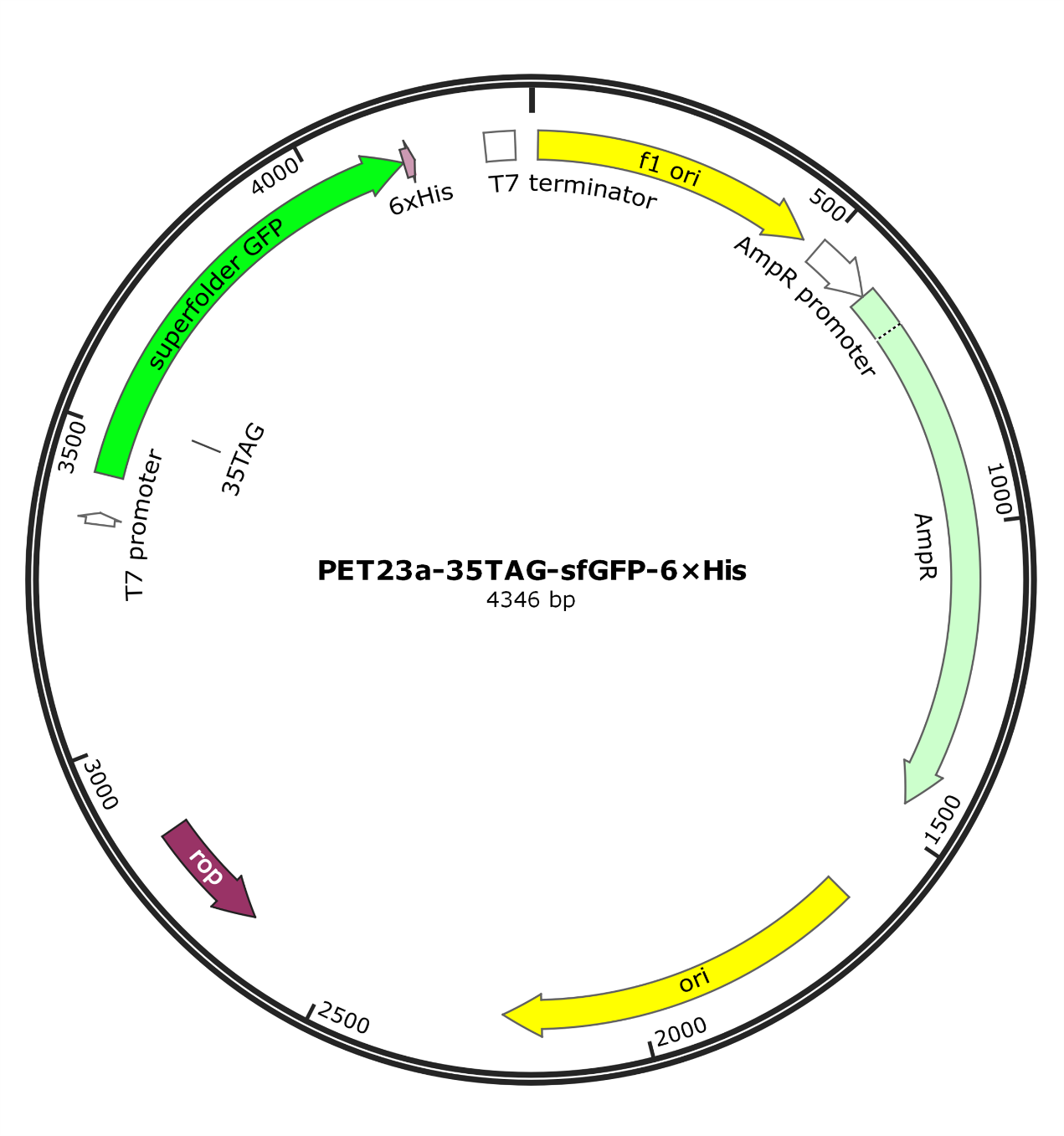


**Fig. S4** The plasmid map of pET23a-35TAG-sfGFP. The 35TAG-sfGFP sequence was located between RBS and T7 terminator on the pET-23a. The codon of 35 site in sfGFP was mutated into TAG. His-tag was at the C-terminus.


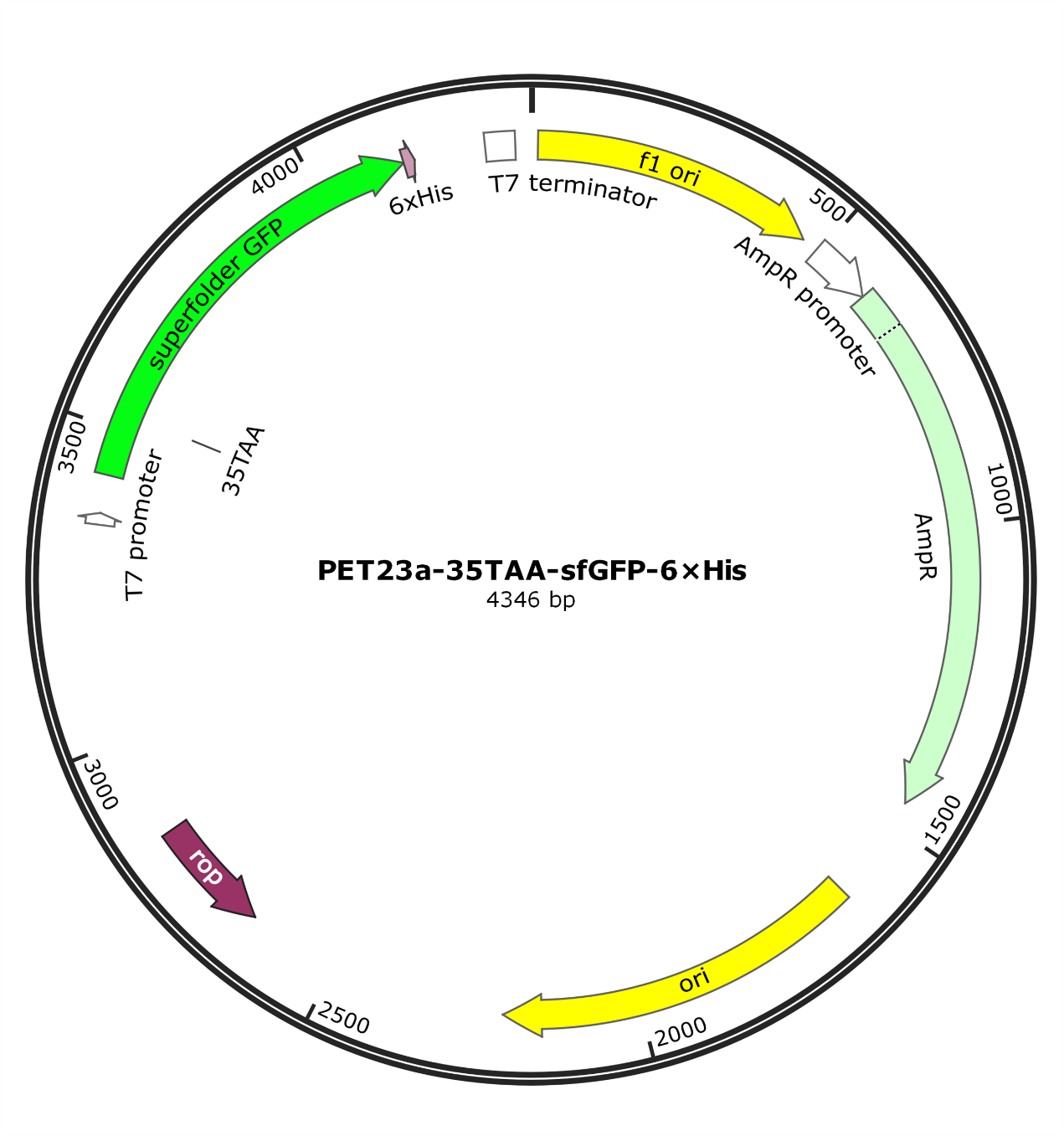


**Fig. S5** The plasmid map of pET23a-35TAA-sfGFP. The 35TAA-sfGFP sequence was located between RBS and T7 terminator on the pET-23a. The codon of 35 site in sfGFP was mutated into TAA. His-tag was at the C-terminus.


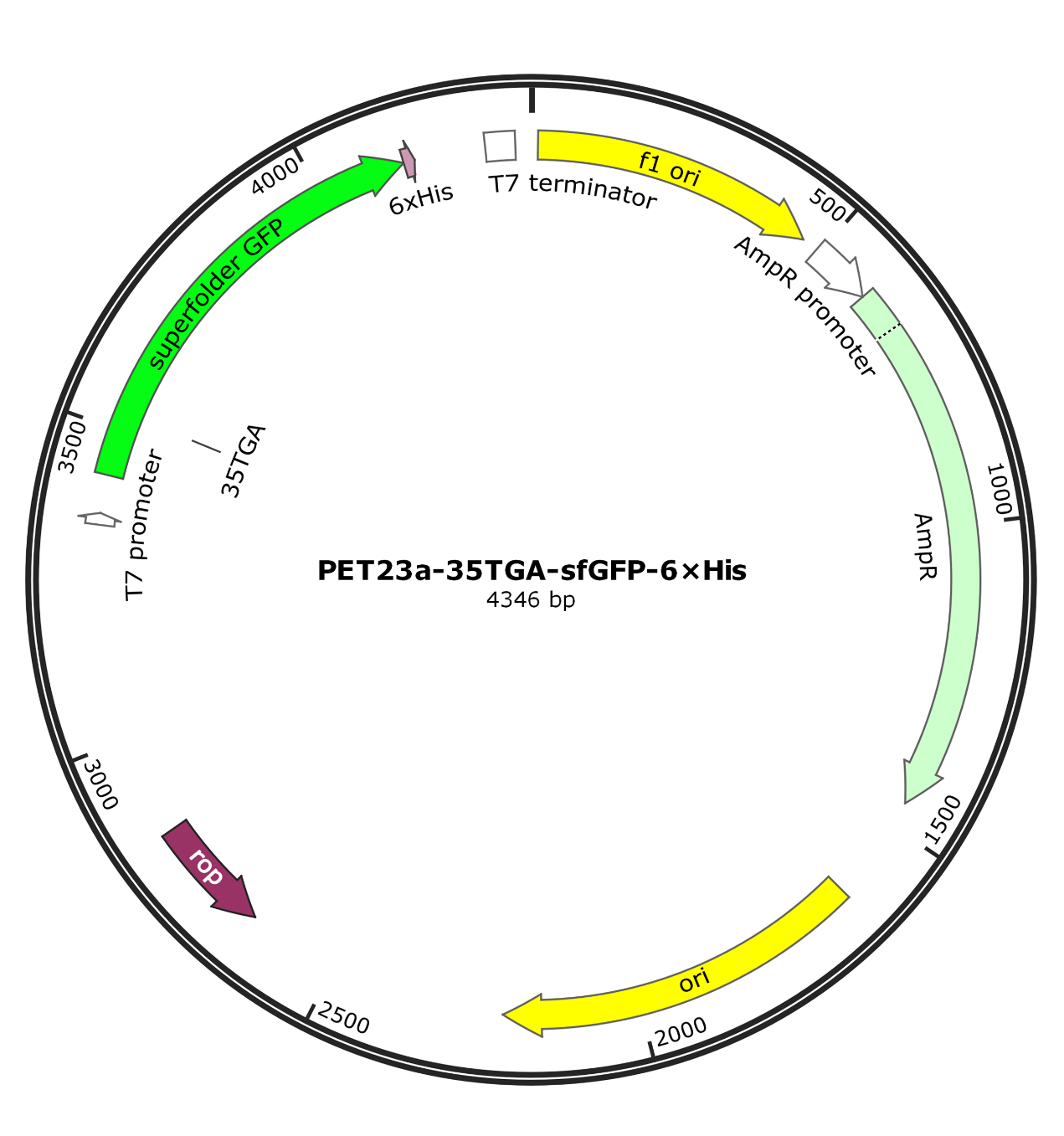


**Fig. S6** The plasmid map of pET23a-35TGA-sfGFP. The 35TGA-sfGFP sequence was located between RBS and T7 terminator on the pET-23a. The codon of 35 site in sfGFP was mutated into TGA. His-tag was at the C-terminus.


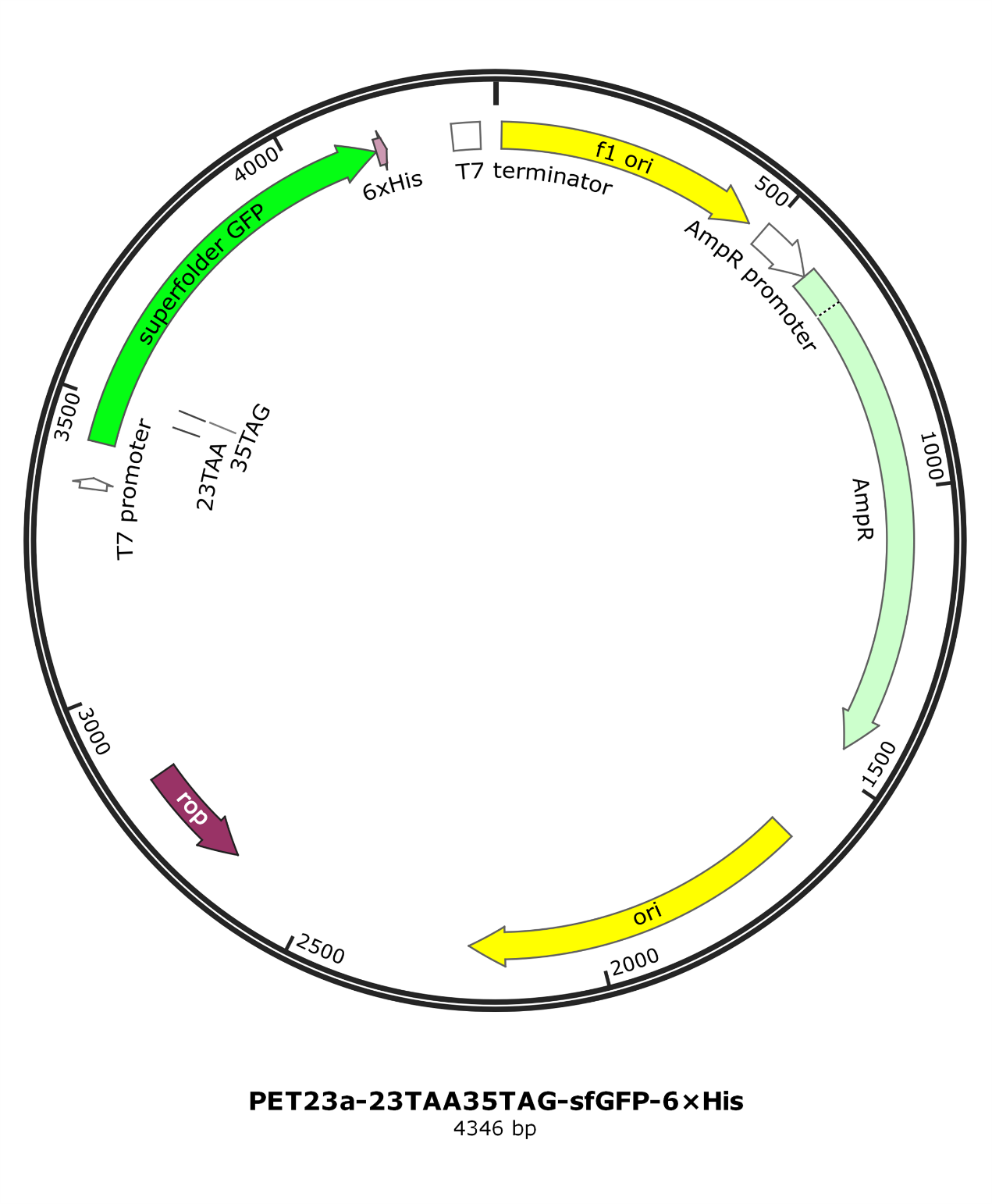


**Fig. S7** The plasmid map of pET23a-23TAA35TAG-sfGFP. The 23TAA35TAG-sfGFP sequence was located between RBS and T7 terminator on the pET-23a. The codons of 23 and 35 sites in sfGFP were mutated into TAA and TAG, respectively. His-tag was at the C-terminus.


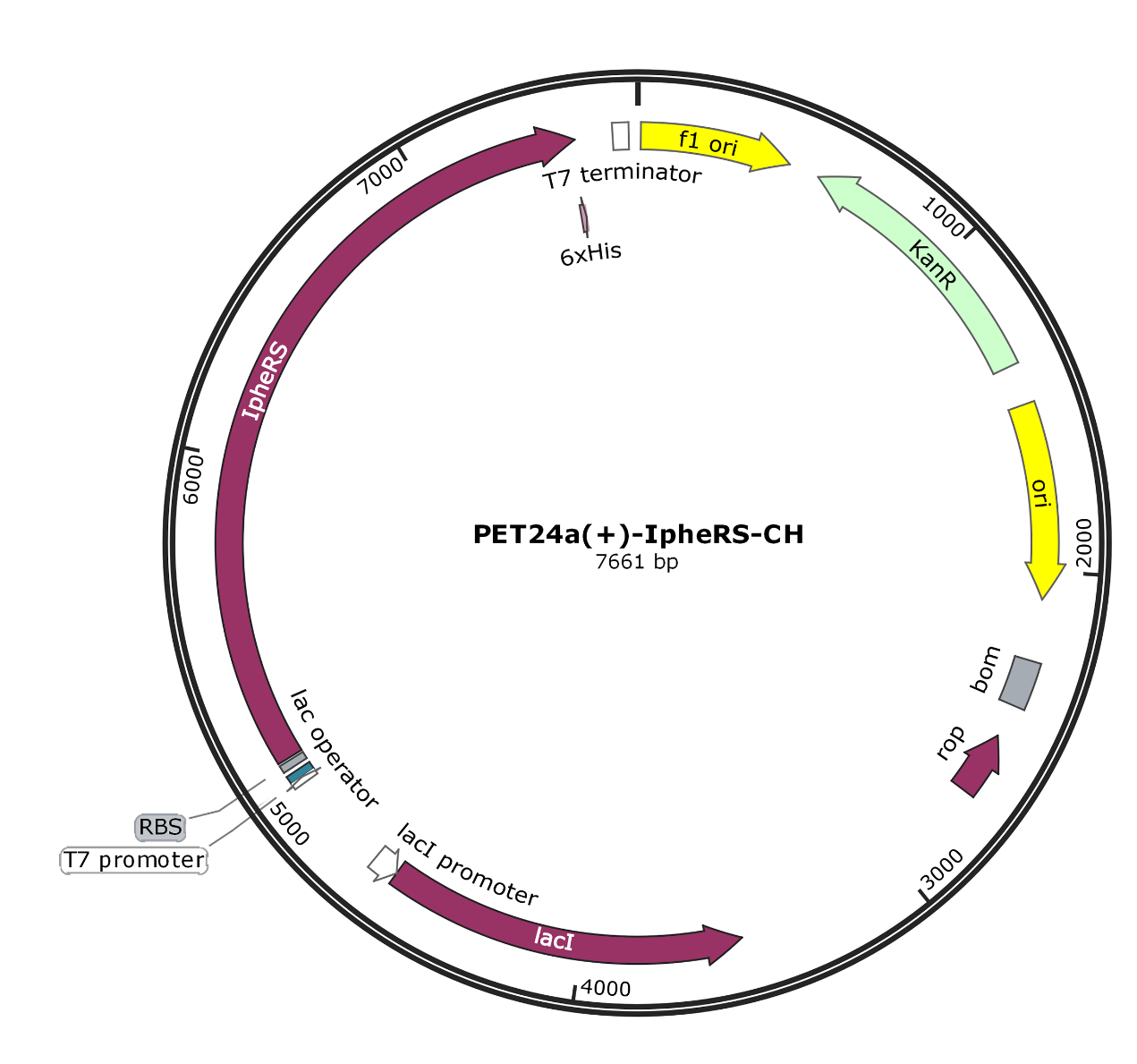


**Fig. S8** The plasmid map of pET24a-IpheRS. The IpheRS sequence was located between RBS and T7 terminator on the pET-24a. His-tag was at the C-terminus.


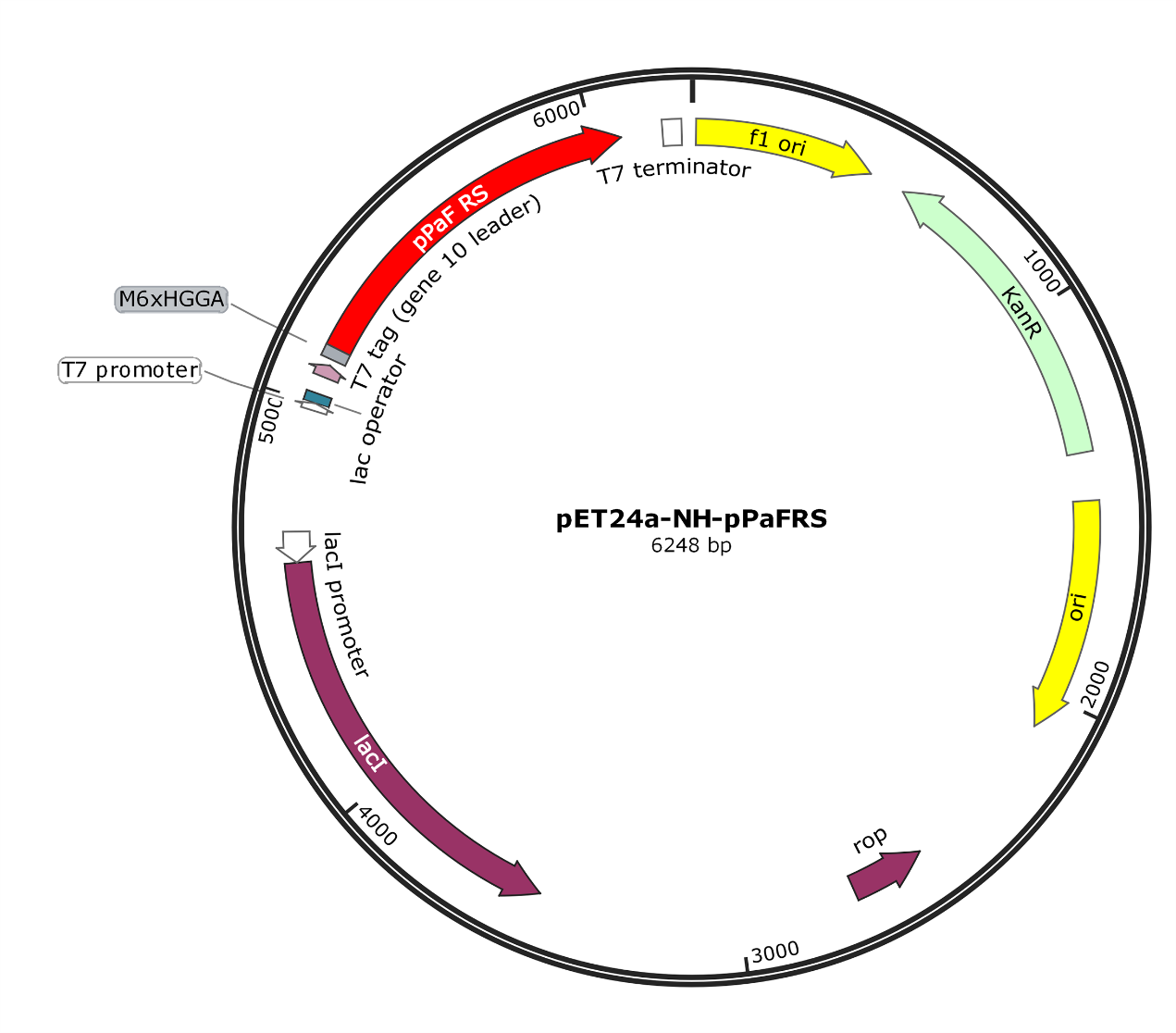


**Fig. S9** The plasmid map of pET24a-pPaFRS. The pPaFRS sequence was located between RBS and T7 terminator on the pET-24a. The His-tag was at the N-terminus.


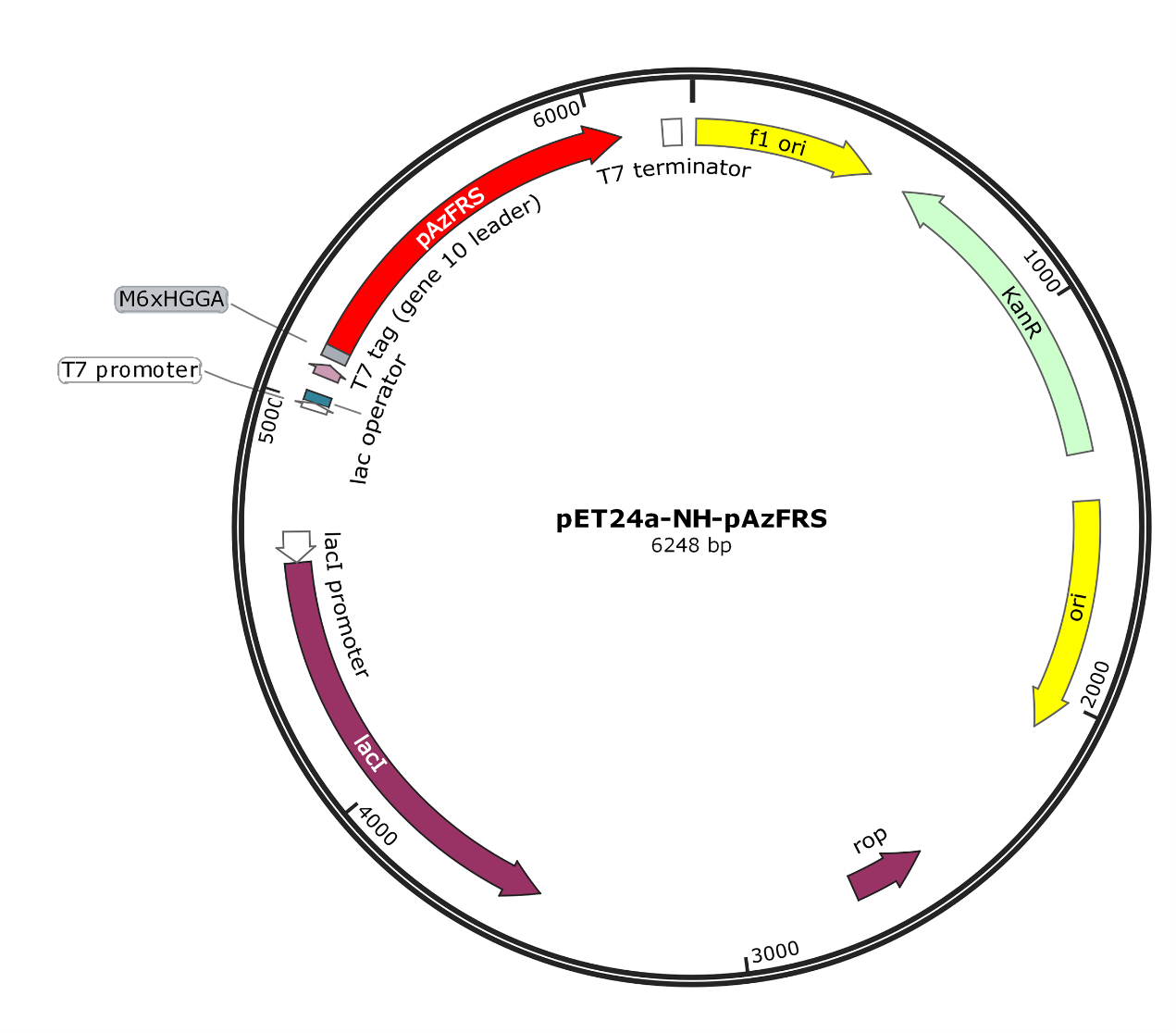


**Fig. S10** The plasmid map of pET24a-pAzFRS. The pAzFRS sequence was located between RBS and T7 terminator on the pET-24a. The His-tag was at the N-terminus.


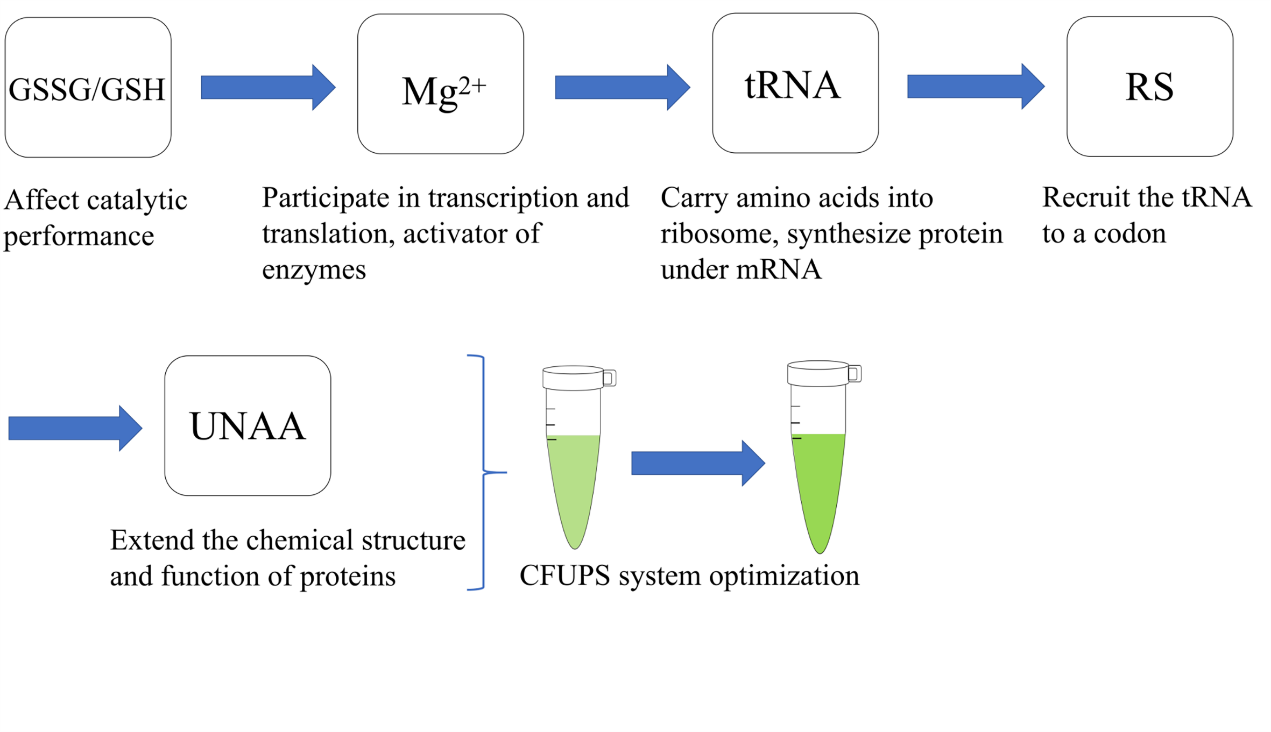


**Fig. S11** Optimization process of reagent components in the system.

**
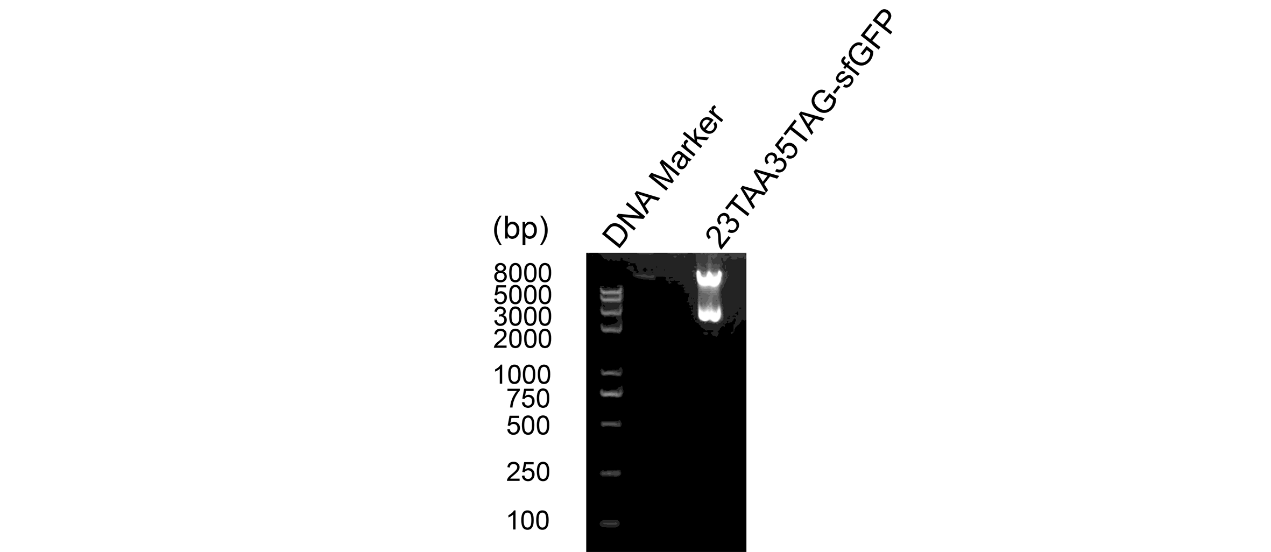
**

**Fig. S12** Electrophoresis results of target protein plasmid. The size of the mutant plasmid 23TAA35TAG-sfGFP was 4346bp, and the agarose gel electrophoresis was performed with 1% DNA gel. The result showed that the band was correct.
